# The inner junction protein CFAP20 functions in motile and non-motile cilia and is critical for vision

**DOI:** 10.1038/s41467-022-33820-w

**Published:** 2022-11-03

**Authors:** Paul W. Chrystal, Nils J. Lambacher, Lance P. Doucette, James Bellingham, Elena R. Schiff, Nicole C. L. Noel, Chunmei Li, Sofia Tsiropoulou, Geoffrey A. Casey, Yi Zhai, Nathan J. Nadolski, Mohammed H. Majumder, Julia Tagoe, Fabiana D’Esposito, Maria Francesca Cordeiro, Susan Downes, Jill Clayton-Smith, Jamie Ellingford, Genomics England Research Consortium, J. C. Ambrose, P. Arumugam, R. Bevers, M. Bleda, F. Boardman-Pretty, C. R. Boustred, H. Brittain, M. A. Brown, M. J. Caulfield, G. C. Chan, A. Giess, J. N. Griffin, A. Hamblin, S. Henderson, T. J. P. Hubbard, R. Jackson, L. J. Jones, D. Kasperaviciute, M. Kayikci, A. Kousathanas, L. Lahnstein, A. Lakey, S. E. A. Leigh, I. U. S. Leong, F. J. Lopez, F. Maleady-Crowe, M. McEntagart, F. Minneci, J. Mitchell, L. Moutsianas, M. Mueller, N. Murugaesu, A. C. Need, P. O’Donovan, C. A. Odhams, C. Patch, D. Perez-Gil, M. B. Pereira, J. Pullinger, T. Rahim, A. Rendon, T. Rogers, K. Savage, K. Sawant, R. H. Scott, A. Siddiq, A. Sieghart, S. C. Smith, A. Sosinsky, A. Stuckey, M. Tanguy, A. L. Taylor Tavares, E. R. A. Thomas, S. R. Thompson, A. Tucci, M. J. Welland, E. Williams, K. Witkowska, S. M. Wood, M. Zarowiecki, Omar A. Mahroo, Jennifer C. Hocking, Michael E. Cheetham, Andrew R. Webster, Gert Jansen, Oliver E. Blacque, W. Ted Allison, Ping Yee Billie Au, Ian M. MacDonald, Gavin Arno, Michel R. Leroux

**Affiliations:** 1 Department of Biological Sciences, University of Alberta, Edmonton, AB Canada; 2 Department of Medical Genetics, University of Alberta, Edmonton, AB Canada; 3 Department of Molecular Biology and Biochemistry, Simon Fraser University, Burnaby, BC Canada; 4 Centre for Cell Biology, Development, and Disease, Simon Fraser University, Burnaby, BC Canada; 5 Department of Ophthalmology & Visual Science, University of Alberta, Edmonton, AB Canada; 6 UCL Institute of Ophthalmology, London, UK; 7 Moorfields Eye Hospital, London, UK; 8 School of Biomolecular and Biomedical Science, Conway Institute, University College Dublin, Belfield, Dublin 4, Ireland; 9 Division of Anatomy, Department of Surgery, University of Alberta, Edmonton, AB Canada; 10 Lethbridge Outreach Genetics Service, Alberta Health Services, Lethbridge, AB Canada; 11 Western Eye Hospital, Imperial College Healthcare NHS Trust, London, UK; 12 ICORG, Imperial College London, London, UK; 13 Oxford Eye Hospital, Oxford University Hospitals NHS Foundation Trust, Oxford, UK; 14 Manchester Centre for Genomic Medicine, Division of Evolution and Genomic Sciences, School of Biological Sciences, Faculty of Biology, Medicine and Health, University of Manchester, Manchester, UK; 15 Manchester Centre for Genomic Medicine, St Mary’s Hospital, Manchester University NHS Foundation Trust, Health Innovation Manchester, Manchester, UK; 16 Division of Evolution and Genomic Sciences, School of Biological Sciences, University of Manchester, Manchester, UK; 17 Genomics England, London, UK; 18 Department of Cell Biology, University of Alberta, Edmonton, AB Canada; 19 Women and Children’s Health Research Institute, University of Alberta, Edmonton, AB Canada; 20 Department of Cell Biology, Erasmus University Medical Centre, Rotterdam, The Netherlands; 21 Department of Medical Genetics, Alberta Children’s Hospital Research Institute, Cumming School of Medicine, University of Calgary, Calgary, AB Canada; 22 North Thames Genomic Laboratory Hub, Great Ormond Street Hospital for Children NHS Foundation Trust, London, UK; 23 William Harvey Research Institute, Queen Mary University of London, London, EC1M 6BQ UK

**Keywords:** Molecular biology, Retinal diseases, Medical genetics, Mechanisms of disease, Hereditary eye disease

## Abstract

Motile and non-motile cilia are associated with mutually-exclusive genetic disorders. Motile cilia propel sperm or extracellular fluids, and their dysfunction causes primary ciliary dyskinesia. Non-motile cilia serve as sensory/signalling antennae on most cell types, and their disruption causes single-organ ciliopathies such as retinopathies or multi-system syndromes. CFAP20 is a ciliopathy candidate known to modulate motile cilia in unicellular eukaryotes. We demonstrate that in zebrafish, *cfap20* is required for motile cilia function, and in *C. elegans*, CFAP-20 maintains the structural integrity of non-motile cilia inner junctions, influencing sensory-dependent signalling and development. Human patients and zebrafish with *CFAP20* mutations both exhibit retinal dystrophy. Hence, CFAP20 functions within a structural/functional hub centered on the inner junction that is shared between motile and non-motile cilia, and is distinct from other ciliopathy-associated domains or macromolecular complexes. Our findings suggest an uncharacterised pathomechanism for retinal dystrophy, and potentially for motile and non-motile ciliopathies in general.

## Abstract (web summary)

Motile and non-motile cilia have distinct functions and protein complexes associated with them. Here, the authors show the conserved protein CFAP20 is important for both motile and non-motile cilia and is distinct from other ciliopathy-associated domains or macromolecular complexes.

## Introduction

**Fig. 1:**
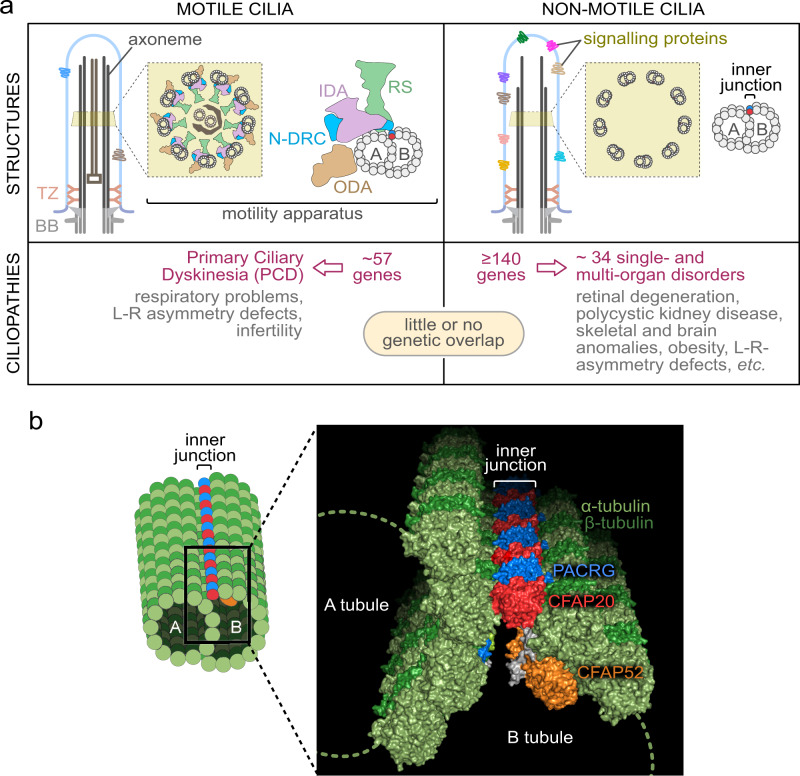
The two principal classes of ciliopathies are considered to be mutually exclusive: they affect the functions of motile or non-motile (primary) cilia, and they have little to no genetic overlap. **a** Functional modules in motile cilia encompass at least 57 proteins, and include subunits of the Inner Dynein Arm (IDA) and Outer Dynein Arm (ODA), which confer motility, as well as subunits within radial spokes (RS) and the Nexin-Dynein Regulatory Complex (N-DRC), which regulate motility. At least 140 proteins are implicated in a wide array non-motile ciliopathies. **b** This study focusses on the protein CFAP20 (red). From reconstructions of motile cilia in *Chlamydomonas* and *Tetrahymena*, CFAP20 is known to form a repeating unit with PACRG (blue); these form the core of the inner junction (IJ) that runs the length of each microtubule doublet. The IJ participates in attachment of the B tubule to the A tubule to complete each of the nine doublet microtubule axoneme. The inner junction structure shown is derived from PDB file 6VE7^21^. TZ transition zone, BB basal body, L-R left-right.

Nearly all eukaryotes, from unicellular organisms to humans, possess motile cilia. The rhythmic beating of these microtubule-based organelles lies at the heart of reproduction, development, and health. Motile cilia enable sperm locomotion, clear respiratory airways, move cerebrospinal fluid, and contribute to left-right body axis patterning^1,2^. Motile cilia are structurally conserved across metazoans, being powered by inner and outer dynein arms, whose coordinated movements are regulated by radial spoke and dynein regulatory complex modules^3^ (Fig. 1a).

Motile cilia disorders in humans, largely synonymous with primary ciliary dyskinesia (PCD), are directly attributable to defects in at least 57 different motility apparatus proteins, or proteins required for their expression or assembly (Fig. 1a). Clinical ailments in PCD include chronic respiratory conditions, infertility, laterality defects, and occasionally hydrocephalus^2^.

Non-motile (or primary) cilia, which lack a motility apparatus, are present on virtually all human cell types. They act as cellular antennae by compartmentalising specialised signal transduction proteins, enabling them to capture and propagate environmental cues^4–6^. For example, cilia in kidney tubules detect the flow and composition of urine, cilia on olfactory neurons transduce odorant signals, and retinal photoreceptors elaborate their cilia to capture photons and enable vision^7–10^.

Human genetics has uncovered approximately 140 proteins known to function in non-motile cilia, and whose disruption causes at least 34 clinically-defined ciliopathies^11–13^ (Fig. 1). Collectively, these ciliopathies are characterised by different progressive and developmental phenotypes, including retinal degeneration, polycystic kidneys, obesity, as well as skeletal and brain malformations.

Based on the above, the genetic underpinnings and clinical phenotypes of motile and non-motile ciliopathies are largely considered mutually exclusive^2,12–14^ (Fig. 1). This assumption has historically been useful in dissecting disease mechanisms; however, with broad access to unbiased genetic analysis such as whole genome sequencing, previously-held rules of gene-disease relationships are being challenged, and scientists must be open-minded to novel hypotheses regarding aetiology. Here, we report findings that challenge the motile *vs*. non-motile ciliopathy dichotomy, by providing evidence that (i) degeneration of photoreceptors (i.e., non-motile cilia) can be caused by disruption of a locus specifically associated with motile cilia function; and (ii) the axoneme inner junction, which connects the B tubule to the A tubule to form each of the doublet microtubules (Fig. 1b), acts as a structural/functional hub, which we term the Inner Junction Hub or IJH, that influences the functions of both motile and non-motile cilia.

Our studies, including the genetics of inherited retinal dystrophy (IRD), led us to focus on an evolutionarily conserved ciliary protein, CFAP20 (Cilia- and Flagella-Associated Protein 20), that is known to regulate ciliary movement (waveform) in *Paramecium* and *Chlamydomonas*, but is under-studied in metazoans^15–18^. Notably, genomes of nematodes such as *C. elegans*, which lack motile cilia altogether, encode CFAP20 (Supplementary Fig. 1). Recent studies position CFAP20 specifically as a core inner junction protein^16,19,20^ (Fig. 1b). Based on near-atomic-resolution cryo-electron microscopy reconstructions of motile cilia axonemes, the inner junction is well positioned to act as a scaffold for the radial spoke and dynein regulatory complex machinery^19–22^. From this ciliary subdomain, we surmised that in motile cilia CFAP20 might help regulate the ciliary waveform, whereas in non-motile cilia CFAP20 could potentially be part of a structural/functional platform (hub) for sensory/signalling modules.

Consistent with this hypothesis, we confirm that zebrafish CFAP20 functions in tissues bearing motile cilia, and plays an essential role in photoreceptor outer segments (i.e., non-motile cilia). In *C. elegans*, CFAP20 is required for the structural integrity of the inner junction and, similar to its protein neighbour PACRG (Parkin Co-Regulated Gene protein), participates in several non-motile cilia-mediated processes, including gustatory plasticity, lifespan control, locomotion and body size determination. Furthermore, we uncovered rare biallelic *CFAP20* missense and canonical splice-site variants in 8 human patients from four unrelated families affected by inherited retinal dystrophy, a vastly heterogeneous condition often caused by non-motile cilia defects.

Together, our study demonstrates unexpected roles for CFAP20 within an unconventional ciliopathy domain or hub centered within the inner junction. Notably, this inner junction hub is distinct from that of other ciliary modules, or macromolecular complexes, that are commonly associated with ciliopathies, and therefore represents an uncharacterised pathomechanism for retinal dystrophy. In humans, several other proteins within or near the IJH—including PACRG—could similarly function in both motile and non-motile cilia, and thus potentially represent additional examples of proteins whose disruption break the dichotomy between motile and non-motile ciliopathies.

## Results

### cfap20 is required for motile cilia in zebrafish

**Fig. 2:**
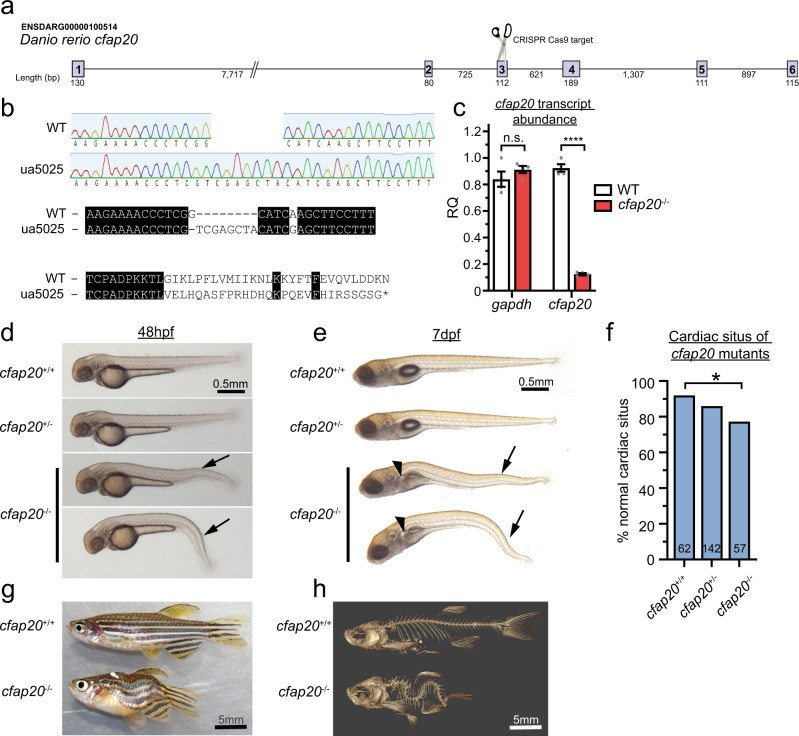
Zebrafish *cfap20* mutants display developmental phenotypes characteristic of a motile ciliopathy. **a** Schematic of CRISPR/Cas9 strategy targeting exon3. **b** The *cfap20*^ua5025^ allele (*cfap20*^*−*^^/−^) has a 1 bp deletion, 8 bp insertion (c.209delGinsTCGAGCTA; p.Gly70Valfs*29) that produces a frameshift at residue 70 and predicts loss of the majority of this deeply conserved protein (see also Supplementary Fig. 1). **c** *cfap20*^*−*^^/−^ mutants have dramatically reduced abundance of transcript compared to wildtype at 48 h post fertilisation (hpf) (*n* = 4, error bars = SEM; two-tailed unpaired T-tests, *gapdh P* = 0.2912; *cfap20 P* = 1.54 * 10^−7^). **d**, **e** *cfap20*^*−*^^/−^ larvae exhibit anterior-posterior body axis kinks/ventral curvature (arrows) displayed at 48 hpf and 7 days post-fertilisation (dpf). **e** *cfap20*^*−*^^/−^ larvae develop pronephric duct cysts (arrowheads) at 7 dpf. **f** *cfap20*^*−*^^/−^ mutants exhibit significantly increased incidence of left-right pattern defects compared to wildtype (cardiac situs, n-value per condition shown, Fisher’s exact test, *P* = 0.0387). **g**, **h** *cfap20*^*−*^^/−^ adult homozygotes develop severe spine curvature compared to age-matched wildtype at 4 months post fertilisation. These motile ciliopathy phenotypes are also observed following knockdown of *cfap20* in zebrafish (see Supplementary Figs. 3, 4). CRISPR clustered regularly interspaced short palindromic repeats, WT wildtype. Source data are provided as a Source Data file.

CFAP20 orthologues have remarkable sequence conservation throughout eukaryotes and are required for motile cilia functions in *Paramecium*, the single-celled algae *Chlamydomonas*, and *Drosophila* sperm^15,17,23^ (Supplementary Fig. 1). Yanagisawa et al. also demonstrated that transient knockdown of *cfap20* disrupts zebrafish left-right organiser cilia, causes cardiac situs defects, and produces body-axis curvature defects^16^. To build upon this observation, and test if CFAP20 is required for motile cilia function in a post-larval vertebrate, we mutated *cfap20* in zebrafish. CRISPR (clustered regularly interspaced short palindromic repeats)/Cas9 was used to produce a frameshift mutation in exon 3 (Fig. 2a, b, denoted allele *cfap20*^ua5025^) that predicts a protein disruption at residue 70 (NM_200811.1:c.209delGinsTCGAGCTA; p.(Gly70Valfs*29)), thereby lacking the majority of the highly conserved CFAP20 residues (Supplementary Fig. 1a). This mutation disrupts the single copy and single splice isoform of *cfap20* identifiable in zebrafish, and we detect no alternative transcripts in *cfap20* homozygotes (Supplementary Fig. 2). The frameshift mutation leads to an ~90% reduction in *cfap20* transcript abundance (Fig. 2c), presumably due to nonsense-mediated decay^24–26^. These data are consistent with *cfap20*^ua5025^ being a null allele or strong hypomorph, and we hereafter refer to the mutants as *cfap20*^−/−^.

To address whether *CFAP20* is required for motile cilia functions in zebrafish, we looked for phenotypes that typically occur in zebrafish larvae and adults when motile cilia are disrupted. Homozygous mutant *cfap20*^*−*^^/−^ zebrafish display various phenotypes that align exactly with those predicted from motile ciliopathies in larval and adult fish. Homozygotes are distinguishable from siblings by anterior-posterior body axis kinks, or ventral body axis curvature at 48 hours post fertilisation (hpf) (Fig. 2d, arrows). Body curvature persists through 7 days post fertilisation (dpf), and pronephric duct cysts develop (Fig. 2e, arrowhead). Mutants also exhibit a significant increase (~15%) in frequency of cardiac situs defects (abnormal left-right axis asymmetry) compared to WT controls (Fig. 2f). Although the severe ventral body curvature phenotype seems incompatible with life, a subset of *cfap20*^*−*^^/−^ homozygotes with kinked body axes survive to adulthood. Adult *cfap20*^*−*^^/−^ homozygotes display severe spinal curvature (Fig. 2g, h), as observed in other zebrafish mutants that model motile ciliopathies^27–31^. The specificity and attribution of these phenotypes to *cfap20* was independently supported via mRNA rescue experiments described below, and via gene knockdown using previously-described morpholino oligonucleotides (MO) that target *cfap20*^16^. Knockdown of *cfap20* induces increasingly penetrant phenotypes in a dose-response format (Supplementary Fig. 3a–c), supporting MO fidelity. Knockdown of *cfap20* closely phenocopies the *cfap20*^*−*^^/−^ mutants, including ventral body curvature and cardiac/gut situs defects at 48 hpf (Supplementary Fig. 4a–f).

Overall, our data demonstrate a requirement for *cfap20* in the function of zebrafish motile cilia. This suggests a deeply conserved function for CFAP20 that has been retained in animals, across diverse tissues, and since its appearance in the last common ancestor of eukaryotes.

### The C. elegans CFAP20 orthologue localises to non-motile cilia independently from PACRG

Considering that a foundational role for CFAP20 in motile cilia is established in unicellular organisms^15,17^, as well as in multiple vertebrate tissues (above), it is notable that this protein also exists in *C. elegans* (Supplementary Fig. 1), despite nematodes not having motile cilia^32^. *C. elegans* uses a diverse range of non-motile cilia to sense its environment and regulate behavioural and developmental processes^32–34^. This led us to speculate that CFAP20 may also function in non-motile cilia. An alternative hypothesis is that the protein has been retained for a non-ciliary function, for example a role at the centrosome and during cell division^35^. We therefore assessed *C. elegans* CFAP20 (termed CFAP-20) for a potential ciliary localisation and function.

**Fig. 3:**
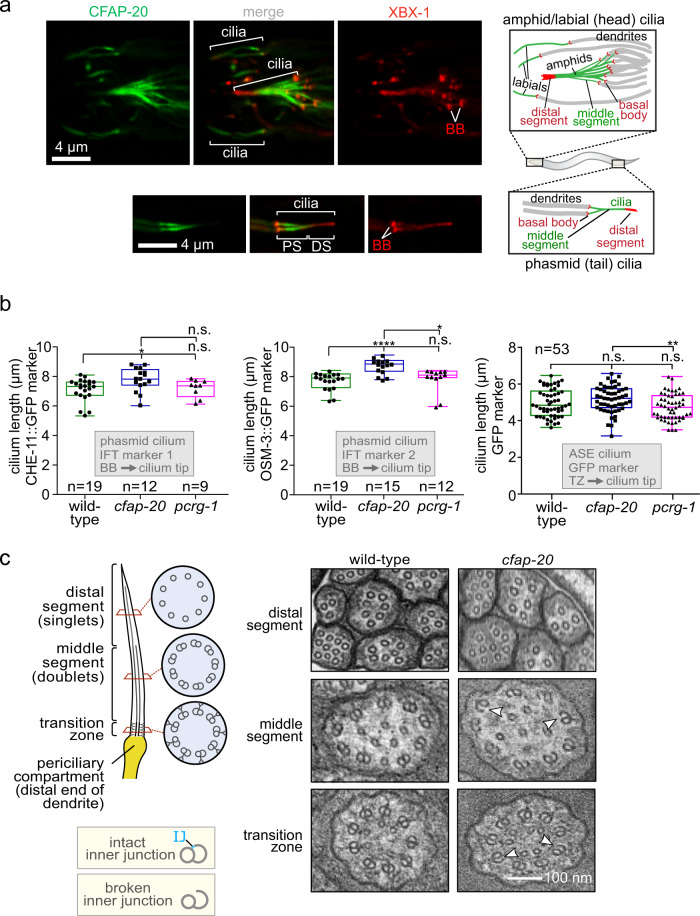
*C. elegans* CFAP-20 localises to non-motile cilia and is required for the structural integrity of the axoneme inner junction. **a** *C. elegans* CFAP-20 is expressed specifically in ciliated sensory neurons and localises to the inner junction-containing proximal segment of the ciliary axoneme. Shown are fluorescence microscopy images of GFP-tagged CFAP-20 (CFAP-20::GFP) localising to cilia in the head (including amphid/labial neurons) and tail (phasmid neurons). Schematics show the relative locations and structural features of different cilia at the dendritic ends of sensory neurons in the head and tail. PS, proximal segment; DS, distal segment. **b** Loss of *C. elegans* CFAP-20 leads to longer phasmid (Kruskal-Wallis and Dunn’s Test; *P* values: CHE-11::GFP: *P* values: WT vs cfap-20 = 0.0186; WT vs pcrg-1 = 0.9999; cfap-20 vs pcrg-1 = 0.1634; OSM-3::GFP: WT vs cfap-20 = 0.0001; WT vs pcrg-1 = 0.6374; cfap-20 vs pcrg-1 = 0.0336) but not ASE cilia (one way ANOVA and Tukey Mann test; GFP (ASE): WT vs cfap-20 = 0.4322; WT vs pcrg-1 = 0.1976; cfap-20 vs pcrg-1 = 0.0097). Graphs represent ciliary length measurements using the cilium-localised CHE-11::GFP and OSM-3::GFP fluorescence reporters in phasmid cilia from wild-type animals and *pcrg-1* mutants, or soluble GFP marker for ASE cilia. Loss of PACRG (*pcrg-1* mutant) does not affect ciliary length. Measurements are from basal body (BB) to ciliary tip for the intraflagellar transport (IFT) markers, and transition zone (TZ) to the ciliary tip for the GFP reporter. Box plots represent minima, 25^th^ percentile, median, 75^th^ percentile, maxima. **c** *C. elegans* CFAP-20 is required for the structural integrity of the axoneme inner junction. The schematic shows normal ciliary/microtubule ultrastructures at the level of the transition zone, middle segment, and distal segment. Transmission Electron Microscopy (TEM) cross-sections of *C. elegans* amphid (head) cilia reveal ultrastructure defects in the *cfap-20* mutant, namely a break at the inner junction (IJ) in transition zone and middle segment doublet microtubules (examples of the defects shown with white arrows). Source data are provided as a Source Data file.

To probe the expression pattern and subcellular localization of CFAP-20, transgenic animals expressing GFP-tagged CFAP-20 under the control of the endogenous *cfap-20* promoter were generated and analysed by fluorescence microscopy. Expression of CFAP-20::GFP is observed only in ciliated cells (Fig. 3a), namely ~60 sensory neurons bearing cilia (mostly located in the head and tail; Supplementary Fig. 5a). CFAP-20::GFP localises within cilia, which are present at the distal ends of dendrites. More specifically, the protein is concentrated in the proximal region of the axoneme, which consists of doublet microtubules, and appears largely absent from the distal region comprised of singlet microtubules (Fig. 3a). This is consistent with this protein localising to the inner junction, where the A and B tubules join to complete the doublet microtubule (Fig. 1b), as reported for *Chlamydomonas*^19–21^.

The high-resolution structures of *Chlamydomonas* axonemes reveal that an alternating arrangement of CFAP20 and another evolutionarily conserved protein, PACRG, make up the core of the inner junction^19–21^. We previously localised *C. elegans* PACRG (PCRG-1) to cilia^36^. Using a *cfap-20* null mutant, generated with CRISPR/Cas9, and a *pcrg-1* null mutant, we show that the localisation of the two proteins is independent of each other (Supplementary Fig. 5b, c).

### C. elegans CFAP-20 is required for the structural integrity of the axoneme inner junction

To assess the potential roles of CFAP20 in non-motile cilia, we subjected our *cfap-20* null mutant to a panel of assays that probe for potential defects in ciliary structures and functions. We compared the *cfap-20* and *pcrg-1* mutants, as we hypothesised that the two juxtaposed proteins might share similar biological functions.

We first assessed the overall structural integrity of *cfap-20* mutant cilia using two established GFP-tagged ciliary markers that are part of the intraflagellar transport (IFT) system which moves along the axoneme (the IFT-kinesin OSM-3/KIF17 and IFT subunit CHE-11/IFT140). Confocal imaging of the fluorescence signals reveals that *cfap-20* mutant cilia are largely intact (Supplementary Fig. 5c). Ciliary markers also enabled accurate measurement of cilia lengths (specifically in tail-situated neurons, which have only two cilia per channel compared to 10 in the head). The *cfap-20* mutant was found to have statistically longer tail cilia (OSM-3 marker, 8.7 ± 0.1 µm; CHE-11 marker, 7.8 ± 0.2 µm) compared to wild-type (7.7 ± 0.1 and 7.1 ± 0.2 µm, respectively), while the *pcrg-1* mutant displayed wild-type-like lengths (Fig. 3b). We measured the length of another type of cilium, from the head-localised ASE neuron, and saw a similar trend—longer *cfap-20* mutant cilium compared to wild-type and *pcrg-1* mutant, but only the latter was statistically significant. This may suggest cell-type specific differences in CFAP-20 ciliary requirements.

To determine if CFAP20 may influence cilium length in mammalian primary cilia, we measured the lengths of cilia following siRNA knockdown of *Cfap20* in mouse NIH3T3 cells. Control cells, transfected with a scrambled *Cfap20* siRNA, showed average cilia lengths of 3.125+/− 0.81 µm (*N* = 6, 222 total cilia counted); in comparison, cells transfected with an siRNA specific to mouse *Cfap20* showed an increase in length, at 3.553+/− 0.83 µm (*N* = 6, 255 total cilia counted) (Supplementary Fig. 6). Hence, cilium length appears to be increased in mammalian *CFAP20* knockdown cells, an observation previously noted in RPE1 cells^23^.

To query the ultrastructure of *C. elegans cfap-20* mutant cilia, we visualised cross-sections of head-localised (amphid) cilia by transmission electron microscopy (TEM). Similar to wild-type animals^37–39^, different sub-compartments within the diverse cilia were observed, namely the transition zone (characterised by Y-links spanning from the doublet microtubule to ciliary membrane), proximal/middle segment made up of doublet microtubules (with A/B tubules), and distal segment, consisting of singlet microtubules with A-tubules only (Fig. 3c). However, in the *cfap-*20 mutant, we observed a large proportion of B-tubules that are open, or unzipped from the A tubules, in both the transition zone and proximal segments of different classes of cilia (Fig. 3c and Supplementary Fig. 7). This seam break phenotype is consistent with a destabilised inner junction, and demonstrates such a role for CFAP20 in metazoans. Notably, disruption of the *Chlamydomonas* CFAP20 orthologue (FAP20) alone does not break the B-to-A tubule connection^20^. In addition, the *C. elegans cfap-20* mutant exhibits structural defects in the specialised mechanosensory cilia of the OLQ neuron (Supplementary Fig. 7) similar to that observed for the *pcrg-1* mutant^36^.

### C. elegans CFAP-20 regulates non-motile cilia-dependent behaviours and development

**Fig. 4:**
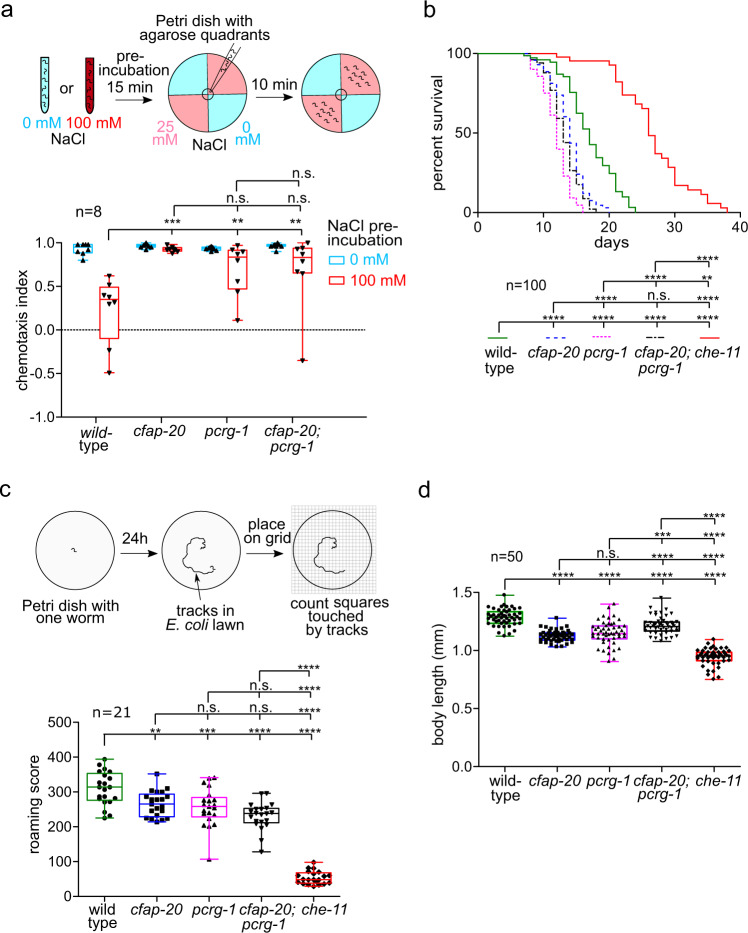
*C. elegans* CFAP20 and PACRG perform non-redundant roles in cilium-dependent behaviours and development: gustatory plasticity, lifespan control, locomotion, and body size. **a** *C. elegans cfap-20* and *pcrg-1* single and double mutants show defects in gustatory plasticity. Wild-type animals pre-exposed to a high NaCl concentration (100 mM) display a reduced attraction to NaCl in chemotaxis assays involving 0 mM and 25 mM NaCl quadrant plates. After exposure to high-NaCl conditions, *cfap-20* and *pcrg-1* single and double mutants still have significantly increased attraction to NaCl compared to wild-type (one-way ANOVA and Bonferroni test *P* values: WT vs cfap-20 = 0.005; WT vs. pcrg-1 = 0.0001; WT vs cfap-20;pcrg-1 = 0.006; cfap-20 vs pcrg-1 = 0.9999; cfap-20 vs cfap-20;pcrg-1 = 0.9999; pcrg-1 vs cfap-20;pcrg-1 = 0.9318). **b** *C. elegans cfap-20* and *pcrg-1* single and double mutants have reduced lifespans. Staged animals are cultured on plates at 20 °C and monitored every 1–2 days for signs of life. Animals with impaired intraflagellar transport and/or prominent cilia structure defects, such as the *che-11* control mutant shown, exhibit an enhanced lifespan. The *cfap-20* single and double mutants (with *pcrg-1*) undergo a statistically-significant reduction in longevity (Log rank Mantle-Cox test; *P* values: WT vs cfap-20 = 0.0001; WT vs pcrg-1 = 0.0001; WT vs cfap-20;pcrg-1 = 0.0001; WT vs che-11 = 0.0001; cfap-20 vs pcrg-1 = 0.0001; cfap-20 vs cfap-20;pcrg-1 = 0.0755; cfap-20 vs che-11 = 0.0001; pcrg-1 vs cfap-20;pcrg-1 = 0.0024; pcrg-1 vs che-11 = 0.0001; cfap-20;pcrg-1 vs che-11 = 0.0001). **c** *C. elegans cfap-20* and *pcrg-1* single and double mutants exhibit a reduced locomotion (roaming defect) common in cilia mutants. The tracks of individual animals are scored with the help of a grid after 24 h. The *che-11* mutant, which has IFT defects and major cilia structure anomalies, is shown together with wild-type as a control (one-way ANOVA and Tukey’s test; *P* values: WT vs cfap-20 = 0.0066; WT vs pcrg-1 = 0.0005; WT vs cfap-20;pcrg-1 = 0.0001; WT vs che-11 = 0.0001; cfap-20 vs pcrg-1 = 0.9426; cfap-20 vs cfap-20;pcrg-1 = 0.0532; cfap-20 vs che-11 = 0.0001; pcrg-1 vs cfap-20;pcrg-1 = 0.2742; pcrg-1 vs che-11 = 0.0001; cfap-20;pcrg-1 vs che-11 = 0.0001). **d** *C. elegans cfap-20* and *pcrg-1* single and double mutants have small body sizes (one-way ANOVA and Tukey’s test; *P* values: WT vs cfap-20 = 0.0001; WT vs pcrg-1 = 0.0001; WT vs cfap-20;pcrg-1 = 0.0001; WT vs che-11 = 0.0001; cfap-20 vs pcrg-1 = 0.4094; cfap-20 vs cfap-20;pcrg-1 = 0.0001; cfap-20 vs che-11 = 0.0001; pcrg-1 vs cfap-20;pcrg-1 = 0.0001; pcrg-1 vs. che-11 = 0.0001; cfap-20;pcrg-1 vs che-11 = 0.0001). The body lengths of staged animals were measured at young adulthood (72 h). All box plots represent minima, 25^th^ percentile, median, 75^th^ percentile, maxima Source data are provided as a Source Data file.

The *C. elegans pcrg-1* mutant can chemotax towards an attractant (NaCl) but exhibits a defect in salt-learning (gustatory plasticity)^36^. We therefore queried if the *cfap-20* mutant behaves similarly. We confirmed that like wild-type worms, *pcrg-1* and *cfap-20* mutants are attracted to NaCl in quadrant assays, being able to distinguish between 0 mM or 1 mM salt (Supplementary Fig. 8a). However, after incubation in a concentrated (100 mM) NaCl solution in the absence of food (bacteria), wild-type worms learn to avoid high salt, whereas both single mutants, and the *pcrg-1;cfap-20* double mutant, remain attracted (Fig. 4a**)**. Such a salt-learning phenotype has been observed in several cilium-associated mutants that impair cilium-dependent G-protein signalling, including *pcrg-1*^36,40–42^.

We then sought to determine if the *cfap-20* mutant also has an altered lifespan, a phenotype ascribed to numerous cilia mutants, including *pcrg-1*, that depends on impaired insulin/IGF1 signalling^36,43,44^. In longevity assays, the *cfap-20* mutant has a reduced lifespan (13.8 ± 0.3 days) compared to wild-type (16.9 ± 0.5 days), similar to the *pcrg-1* mutant and the *cfap-20;pcrg-1* double mutant (Fig. 4b**)**. Notably, such a reduction in lifespan is not due to the overall health of the mutant animals and is markedly different from the long-lifespan phenotype of cilia mutants that impair intraflagellar transport, which is required to build the ciliary axoneme^36,43–47^. Indeed, we specifically tested for IFT system behaviour in the *cfap-20* mutant and found that the movement of particles along the axoneme in the anterograde (base of cilium to tip) and retrograde (tip to base) directions is not greatly different from wild-type (Supplementary Fig. 8b).

Given the shared roles of CFAP-20 and PCRG-1 in phenotypes associated with cilia (gustatory plasticity and altered lifespan), we further sought to determine if these proteins might influence an additional, well-documented cilium-dependent behaviour—locomotion. Specifically, *C. elegans* movement alternates between a slow-moving (dwelling) state in the presence of food, and a more rapid food-searching (roaming) state^48^. Similar to various cilia mutants, both the *cfap-20* and *pcrg-1* single mutants and *cfap-20;pcrg-1* double mutant exhibit reduced roaming, covering a smaller area of the assay plate (Fig. 4c).

Lastly, some cilia mutants display an altered body length, which depends on different signalling pathways, including cGMP and TGF-β^48–51^. The lengths of the *cfap-20* and *pcrg-1* single mutant animals were measured and found to be statistically shorter compared to wild-type (as did the *cfap-20;pcrg-1* double mutant), although not as short as an IFT mutant (*che-11*) which largely lacks a ciliary axoneme (Fig. 4d).

In summary, our findings reveal that *C. elegans* CFAP-20 performs essential roles in non-motile cilium-dependent physiological and developmental processes, namely gustatory plasticity, roaming, lifespan, and body size determination. PCRG-1, its known neighbour within the inner junction, localises independently of CFAP-20 but performs the same cilium-associated functions, potentially acting in the same pathway. Because *C. elegans* lack motile cilia, we conclude that both inner junction proteins have shared but non-redundant, sensory-related functions in non-motile cilia.

### Identification of biallelic CFAP20 mutations in patients with inherited retinal dystrophy (IRD)

Independently, we investigated the genetic aetiology of IRD in otherwise genetically undiagnosed individuals. Notably, IRD represents a common clinical phenotype in syndromic and non-syndromic non-motile ciliopathies^9,12,14^. Through exome and genome analysis at two genetics centres (Moorfields/UCL, UK and University of Calgary, Alberta, Canada), biallelic variants were identified in *CFAP20* as candidates for disease causality.

**Fig. 5:**
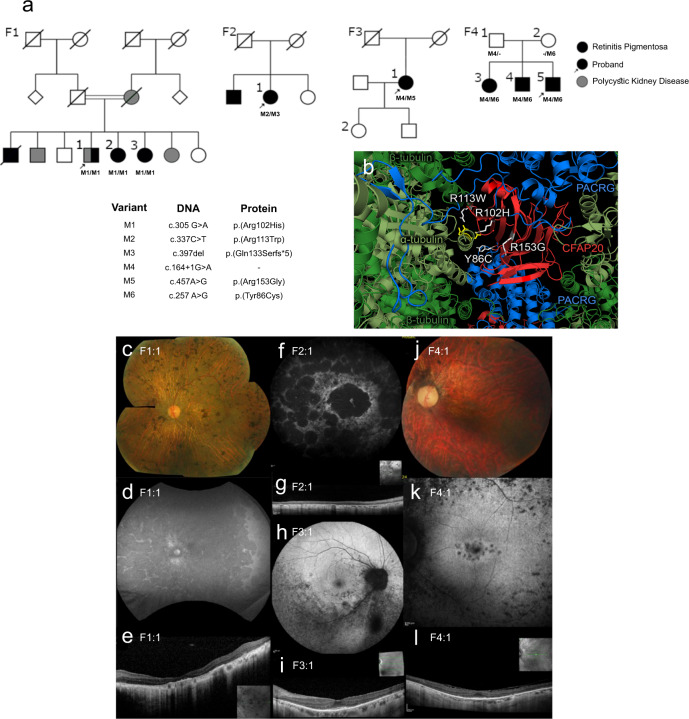
*CFAP20* variants segregate with autosomal recessive retinal dystrophy. **a** Four families (F1 through F4) presenting with early-onset, progressive Retinitis Pigmentosa (RP) from two Ophthalmology Centres (UK & Canada). In each pedigree, biallelic variants in *CFAP20* (M1 thru M6) segregate with RP. Family 1 also exhibits polycystic kidney disease that segregates separately from RP and *CFAP20*. Arrow indicates proband in each pedigree (Details of symptoms are in Table 1 and summarized in Supplementary Table 3; further details of *CFAP20* gene variants are in Supplementary Table 2). **b** The residues altered in each of the four patient variants (white) are predicted to alter residues in the CFAP20 protein (red) that are on the surface of the protein and therefore may impact upon interactions with PACRG (blue). The variants likely alter key components of the microtubule inner junction (see Fig. 1b for context). Protein structure derived from PDB file 6VE7^21^. Colour fundus photographs of the left eye (**c**, **j**; F1:1 and F4:1 respectively) show a generalized retinopathy with peripheral pigment deposition, attenuated retinal vasculature and marked chorioretinal atrophy (**c**) and peripapillary atrophy (**j**). Autofluorescence imaging (**d**, **f**, **h**, **k**; F1:1, F2:1, F3:1, F4:1 respectively) demonstrates widespread decreased autofluorescence (**d**, **f**) indicating significant atrophy of retinal pigment epithelium (RPE) and focal lesions of RPE atrophy at the macula and midperiphery (**k**) and predominantly the inferior retina (**h**). Optical coherence tomographic imaging (**e**, **g**, **i**, **l**; F1:1, F2:1, F3:1, F4:1, respectively) shows significant loss of outer retinal layers (**e**, **g**) with some surviving outer retina at the fovea (**i**, **l**).

We describe eight individuals from four independent families with damaging biallelic variants in *CFAP20* that segregate with retinal dystrophy (Fig. 5a and Supplementary Table 2). All these variants interestingly cluster to one side of the protein, and two of the residues directly contact α-tubulin (Fig. 5b). Pedigrees and genetic findings are summarised in Fig. 5 and clinical descriptions can be found in Table 1 and Supplementary Table 3. Exome sequencing/Genome Sequencing (ES/WGS) studies of the families revealed homozygous or compound heterozygous variants in *CFAP20* (NM_013242) segregating with the retinal phenotype. Individual F1:1 (GC17761) is a 65-year-old male of Sudanese descent born to consanguineous (2^nd^ cousin) parents (Fig. 5c–e). He has two similarly affected sisters (F1:2, and F1:3). He also has an independent, maternally inherited, polycystic kidney disease (PKD) that does not segregate with the retinal dystrophy in the family (Fig. 5a). Autozygosity mapping analysis and exome sequencing of the three affected siblings identified 2 rare homozygous variants within a 25 Mb autozygous region spanning chr16q21: (hg19) chr16:66,776,473C > T, *DYNC1LI2* NM_006141:c.397G > A p.(Val133Ile) and chr16:58,149,333C > T, *CFAP20* NM_013242:c.305G > A p.(Arg102His). The *DYNC1LI2* variant was predicted to be possibly damaging and tolerated by Polyphen2 and SIFT respectively and is seen in 4 alleles (0.000016 MAF) in the gnomAD v2.1.1 dataset^52^, the *CFAP20* variant was predicted to be probably damaging, and deleterious and is seen in 3 alleles (0.000011 MAF) in the gnomAD dataset. No additional candidate *DYNC1LI2* variants were observed in the 100KGP retinal dystrophy cohort. Therefore, we prioritised *CFAP20* as the most promising candidate.

**Table 1:** Summary of clinical observations

Family ID	Individual ID	Age at examination	BCVA	Fundus findings and ocular history	OCT	ERG	Non-ocular findings
F1	1	65	PL OD, PL OS	Nyctalopia (age 18), peripheral and central vision loss. RPE atrophy, bone spicules, vessel attenuation.	Outer retina/RPE loss	pERG: undetectable, ffERG: undetectable (scotopic/photopic)	PKD (did not segregate with RP)
F2	1	42	N/A	RPE/outer retina atrophy.	Outer retina/RPE loss	N/A	None
F3	1	70	20/20 OD, 20/20 OS	Nyctalopia, superior field loss, inferior and inferio-temporal intra-retinal pigment deposition.	Atrophy of outer retinal layers with relative preservation of central retina	ffERG:delay/amplitude reduction (scotopic), a-wave delay, b-wave reduction/delay (photopic)	None
F4	3	35	20/40 OD, 20/20 OS	Right eye ambylopia and anisometropia.	–	ffERG: No recordable photopic or scotopic responses.	Gross and fine MD, walked at 18 months. Mild hypertonia and brisk reflexes in lower limbs. Learning disability. Premature ovarian insufficiency.
	4	33	20/40 OU	Mild myopia (−2.25 OU). III4e visual field central 10^o^. Bone spicule pigment clumping in periphery. Unmasking of the large choroidal vessels in the mid-periphery; prominent arteriolar attenuation.	Atrophy of outer retinal layers with relative preservation of central retina.	ffERG small residual cone-driven responses to flash and flicker stimuli under photopic conditions. No rod driven responses	Gross and fine MD, walked at 17 months. Spastic diplegia, dystonic hand posturing with ambulation. Learning disability. MRI brain shows mild volume loss, mild thinning of CC, and non- specific T2 hyperintensities. Low spermatozoa concentration, motility unable to be assessed.
	5	31	20/200 OD; 20/70 OS	Laser surgery to correct high myopia (−11OD; −13OS) now Mild myopia (−1.5 OD; −2.75 OS). III4e central 20 degrees.	–	Reduced rod responses	Mild gross MD. Learning disability and visual processing disability, ADHD. Tonic clonic seizures onset at 19 years. MRI normal with mild signal asymmetry in cortical spinal tract. Low spermatozoa concentration with impaired motility.

*ADHD* Attention Deficit Hyperactivity Disorder, *BCVA* Best-corrected Visual Acuity, *CC* Corpus Callosum, *ERG* Electroretinogram (*p* pattern, *ff* full field), *MD* Motor Delay, *MRI* Magnetic Resonance Imaging, *OCT* Optical Coherence Tomography, *OD* Right eye, *OS* Left eye, *OU* Both eyes, *PKD* Polycystic Kidney Disease, *PRs* Photoreceptors, *RPE* Retinal Pigment Epithelium.

Family 2 comprises 3 siblings of Spanish descent, 2 of whom have a retinal dystrophy though only one, a 42-year-old female, contributed DNA to this study (Fig. 5f, g). She harbours compound heterozygous variants in *CFAP20*, c.337C > T: p.(Arg113Trp); c.397delC: p.(Gln133Serfs*5).

A single affected member of family 3 underwent WGS, a 70-year-old female with sectorial RP and peripheral vision loss (Fig. 5h, i). WGS revealed she was compound heterozygous for CFAP20 c.164 + 1G > A, and c.457A > G:p.(Arg153Gly). Finally, Family 4 comprises 3 affected siblings with a generalized retinopathy (Fig. 5j–l) with variable neurological deficits (learning disabilities, poor motor coordination and delayed early motor milestones) and reproductive issues (low sperm motility or ovarian insufficiency). ES analyses identified that all 3 siblings were compound heterozygous for CFAP20 c.164 + 1G > A and c.257G > A:p.(Tyr86Cys) For all families described, no individuals have had signs or symptoms of PCD such as chronic or unusual respiratory symptoms, and unless otherwise specified there has been no history of renal disease. However, not all individuals have had echocardiogram or imaging of kidneys.

### Mutations in CFAP20 lead to reduced protein stability and functionality

**Fig. 6:**
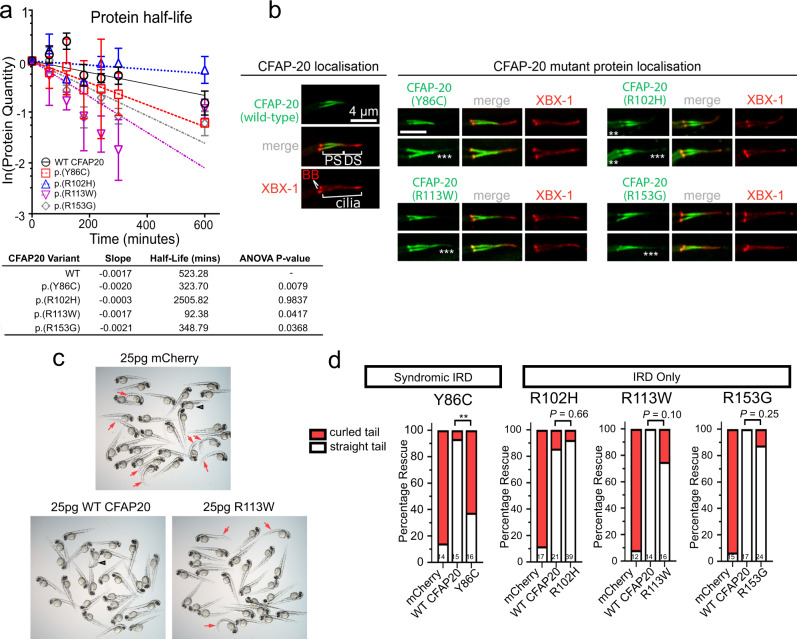
*CFAP20* patient variant sequences variously cause altered protein stability, mislocalisation in *C. elegans* and reduced ability to rescue zebrafish homozygote development. **a** Half life calculations assuming first-order decay (ln([relative protein]*t* = *x*/[relative protein]*t* = 0)). Lysates from transfected HEK293T cells revealed a significant (one-way ANOVA) decay for all variants, except for p.(Arg102His), compared with Wildtype (WT) protein (half-life of ~523 min). The fastest decay was variant p.(Arg113Trp) with a half-life of ~93 min (*n* = 3; error bars = SEM). **b** In *C. elegans*, human *CFAP20* patient variants mislocalise in comparison to wildtype *CFAP20*. The GFP-tagged CFAP20 proteins bearing patient mutations concentrate mostly to the proximal segment (PS) of the axoneme, which is similar to wild-type *CFAP20* protein expressed in *C. elegans*. However, patient variants also mislocalise (asterisks) in the distal segment (DS) and/or the dendritic region to the left of the phasmid cilia (especially p.(Arg102His)). XBX-1 is an IFT reporter that marks the basal body (BB) and entire axoneme. **c** Wildtype human *CFAP20* mRNA rescues zebrafish *cfap20* mutants, as compared to control mRNA (mCherry) or *CFAP20* mRNA bearing patient variants. A representative *cfap20*^+/−^ incross is displayed, injected with WT/patient variant (p.(Arg113Trp)) *CFAP20*, or control mRNA. Mendelian ratios of 25% body axis curvature (red arrows) were expected where treatments failed to rescue the *cfap20*^−/−^ homozygotes. Larvae displaying mRNA toxicity phenotypes (i.e. oedema/dorsalization) occur rarely (black arrowhead). **d** The frequency of rescued (straight tail) and not rescued (curled tail) *cfap20*^−/−^ homozygotes were quantified for each missense human variant. p.(Tyr86Cys), which is associated with syndromic inherited retinal disease (IRD), failed to rescue body curvature. Conversely, the p.(Arg102His), p.(Arg113Trp), and p.(Arg153Gly) variants, that we associated with non-syndromic IRD, are not significantly different from WT *CFAP20* mRNA (Fisher’s exact test; p.(Tyr86Cys) *P* = 0.0021; sample sizes, i.e. the number of *cfap20*^−/−^ homozygous mutant larvae, are indicated at the bottom of each bar). See also Supplementary Fig. 8. Source data are provided as a Source Data file.

Since all the identified *CFAP20* variants were predicted to be deleterious (Supplementary Table 2), we commenced assays to test protein stability and functionality. First, CFAP20 protein variant degradation was monitored over time in cycloheximide-chase experiments. Wild-type and variant CFAP20 proteins bearing a C-terminal*myc* tag were overexpressed in HEK293T cells, and their abundance quantified over a period of 10 h. Analyses showed a statistically significant decline of mutated CFAP20 protein levels after 10 h, except for the p.(Arg102His) variant, compared to WT control. Interestingly, p.(Arg113Lys) shows a sharp decline at an earlier time point (Fig. 6a**)**.

To determine if the patient variants affect the ability of CFAP20 to correctly localise to cilia, we took advantage of the fact that the amino acids in question are identical between the *C. elegans* and human CFAP20 orthologues (Supplementary Fig. 1a). We, therefore, generated strains that express GFP-tagged *C. elegans* CFAP-20 proteins bearing the individual mutations (p.(Tyr86Cys); p.(Arg102His); p.(Arg113Trp); p.(Arg153Gly) variants) and visualised these by confocal microscopy. Remarkably, all four variant proteins still localise to the ciliary axoneme and are concentrated within the proximal region containing doublet microtubules and inner junction (Fig. 6b). However, the localisation patterns do deviate from wild-type, as all proteins display partial mislocalisation (indicated by asterisks) to the distal region of the axoneme, and/or to the dendrite.

Finally, to determine if CFAP20 patient variants have reduced function we assessed their ability to rescue motile ciliopathy phenotypes in zebrafish. We expected delivery of wildtype *CFAP20* mRNA to rescue the fully penetrant body axis phenotypes due to the high conservation of the gene. During early development, motile cilia produce cerebrospinal fluid flow that is required for normal body axis extension during early zebrafish development^27,31^. Embryos generated from heterozygous *cfap20*^+/−^ incrosses were injected with zebrafish *cfap20* or human wildtype *CFAP20* mRNA at various concentrations and scored for the body axis curvature defects. Both zebrafish and human *CFAP20* homologs rescued the expected 25% frequency of phenotypes, including low doses of human mRNA (25 pg), whereas control mRNA did not. (Supplementary Fig. 9, Fig. 6c). Large doses of *CFAP20* mRNA exhibited toxicity, but doses of 25–200 pg mRNA rescued axis curvature with <10% mRNA toxicity (Supplementary Fig. 9b, c). Human *CFAP20* patient variants p.(Arg102His), p.(Arg113Trp) and p.(Arg153Gly), that associated with human IRD only, rescued to a degree that was not significantly different compared to wildtype human *CFAP20* (Fig. 6d); although the p.(Arg113Trp) variant did appear to rescue to a lesser degree than WT and was approaching significance (*P* = 0.10) with our modest sample sizes (Fig. 6c, d). However, the p.(Tyr86Cys) variant (associated with IRD and extraocular phenotypes) was unable to rescue *cfap20*^−/−^ homozygotes compared to wildtype human *CFAP20* (Fig. 6d). These data suggest that the p.(Tyr86Cys) mutation may be more damaging to function than the other variants, at least in the context of motile cilia.

### Loss of zebrafish cfap20 leads to progressive photoreceptor dystrophy and functional deficit of the retina

Patient data revealed that biallelic *CFAP20* variants segregate with retinal dystrophy. To support that this represents a causal relationship, we sought to determine if disrupting *cfap20* causes retinal degeneration in zebrafish. Ocular development was grossly normal in *cfap20*^−/−^ homozygotes at 7dpf, including presence of all neural retinal layers (Supplementary Fig. 10a). Upon quantification it was noted that *cfap20*^−/−^ homozygotes had a slight reduction in globe diameter compared to controls (~9% reduction, *P* < 0.0001, Supplementary Fig. 10b). Since CFAP20 is present within the photoreceptor cilium^53^ we next examined photoreceptor morphology in rod (Tg(−3.7rho:EGFP)kj2) and cone (Tg(−5.5opn1sw1:EGFP)kj9) reporter transgenic lines. At 7 dpf the rod photoreceptors of *cfap20*^−/−^ homozygotes were indistinguishable from controls, whereas cone length was reduced by ~19% (*P* = 0.012; Supplementary Fig. 10c–f). These retinal phenotypes are mild, but we cannot discount transcriptional adaptation^54,55^, or maternal mRNA inheritance (Supplementary Fig. 11) from obscuring a requirement for *cfap20* in early development.

**Fig. 7:**
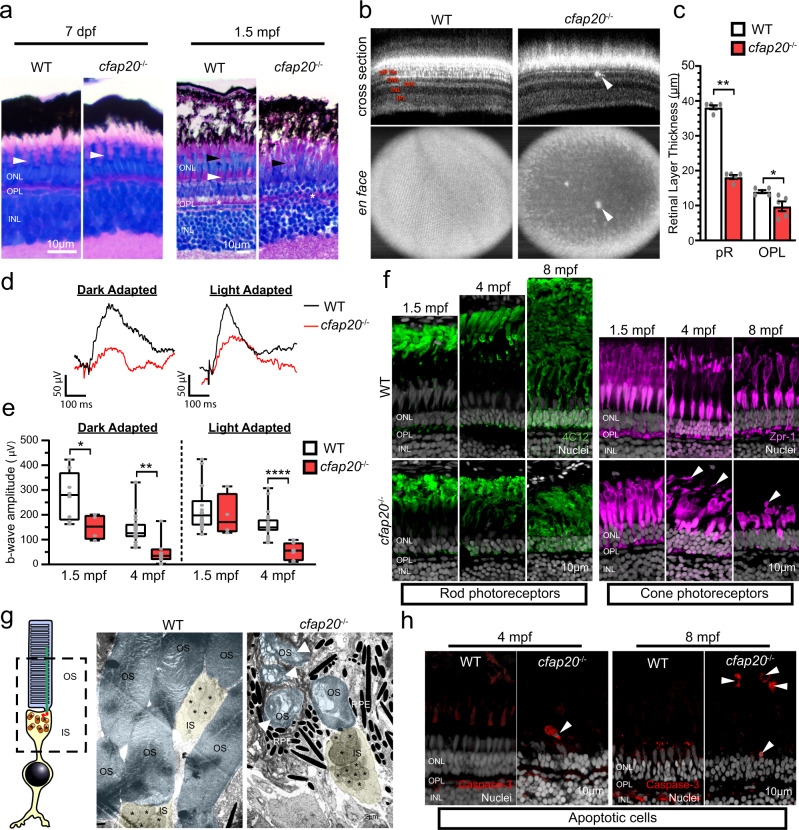
Zebrafish *cfap20*^−/−^ mutants model early-onset, progressive retinal dystrophy. **a** The larval, 7 days post-fertilisation, *cfap20*^−/−^ homozygote retina is indistinguishable from wildtype (WT) animals (green bracket = rod and blue/red/green cone OS and RPE, white arrowhead = UV cone OS). By 1.5 months post-fertilisation (mpf) the homozygote retina is degenerate with loss of organised cone subtype layers (green bracket = rod OS and RPE, white arrowhead = UV cone OS, black arrowhead = blue/red/green cone OS) and poorly defined OPL (asterisk). **b, c** By 4 mpf, the cone mosaic is lost in homozygotes and hyperreflective blebs (arrows) are observed in the outer retina (Optical Coherent Tomography B-scans (cross section) and *en face* projections). The photoreceptor and OPLs are thinner in homozygotes (*n* = 5; error bars = SEM; two-tailed unpaired Mann-Whitney, pR *P* = 0.0079, OPL *P* = 0.0238). Representative full-field electroretinogram traces from 4 mpf dark (DA) or light adapted (LA) animals presented in **d**. **e** At both ages, the DA b-wave is reduced in homozygotes (two-tailed unpaired T-tests; 1.5 mpf: *n* = 8 WT, 4 *cfap20*^−/−^; *P* = 0.0382; 4 mpf *n* = 19 WT, 7 *cfap20*^−/−^; *P* = 0.0017) whereas the LA b-wave is reduced only at 4 mpf (two-tailed unpaired T-tests; 1.5 mpf: *n* = 14 WT, 4 *cfap20*^−/−^; *P* = 0.6010; 4 mpf: *n* = 20 WT, 7 *cfap20*^*−*^^/−^; *P* = 5.60 * 10^−5^). Box plots: minima, 25^th^ percentile, median, 75^th^ percentile, maxima. **f** Rod and cone layers are shorter and became disorganised over time, with cone OSs detachment at 4 and 8 mpf (arrowheads). **g** False-coloured TEM micrographs reveal reduced density and OS dysmorphia of homozygotes at 8 mpf. Detached OSs (arrows) are engulfed by RPE. **h** Apoptotic cells (arrowheads) are observed in the outer retina of homozygotes but never WTs controls. IPL inner plexiform layer, INL inner nuclear layer, OPL outer plexiform layer, ONL outer nuclear layer, pR photoreceptor layer, IS inner segment, OS outer segment, RPE retinal pigment epithelium. See also Supplementary Figs. 9 and 10. Source data are provided as a Source Data file.

Because human *CFAP20* patients first presented with visual deficit between adolescence and adulthood (17–56 years old), we also examined retinal histology in our zebrafish model at older timepoints. At 1.5 months post fertilisation (mpf) there was clear retinal disorganisation visible in *cfap20*^−/−^ homozygote histological samples. The rod outer segment/RPE layer was visibly thinner (green bracket Fig. 7a) and there was very poor definition of the separate UV cone and blue/red/green cone layer (black and white arrowheads respectively, Fig. 7a), suggestive of disorganisation. This disorganisation was not detected in younger animals, i.e. 7dpf, via TEM (Supplementary Fig. 10g).

In young adult fish at 4 mpf, we were able to perform optical coherence tomography (OCT), a key diagnostic test in human retinal dystrophy. At this age, *en face* projections revealed an almost complete abolition of the ordered photoreceptor mosaic organisation present in WT animals (Fig. 7b). Hyperreflective foci were also detected within the outer retinal layers (Fig. 7b, arrow), which may represent activated immune cell infiltration, consistent with retinal degeneration^56–58^. From OCT projections we were also able to determine that photoreceptor and outer plexiform layer thicknesses were substantially reduced in *cfap20*^−/−^ homozygotes (~50% and 30% compared to WT; *P* = 0.0079, *P* = 0.0238 respectively Fig. 7c).

Having identified a potential retinal dystrophy phenotype in *cfap20*^−/−^ homozygotes, we then performed a full-field electroretinogram (ffERG) analysis to quantify the visual function of mutants. ffERG is a key diagnostic tool to measure retinal neuron activity in response to light stimuli (*e.g*., patients with *CFAP20* variants had ERG deficits; Table 1 and Supplementary Table 3). Under both dark-adapted (mixed rod and cone response) and light-adapted (cone-dominated response) conditions, *cfap20*^-/-^ homozygote zebrafish at 4 mpf consistently had dampened responses compared to controls (Fig. 7d).

The large, positive b-wave generated by ON bipolar cell depolarisation^59–61^ is downstream of photoreceptor synapses and requires functional photoreceptors and interneuron health to elicit a normal response. Amplitude of the b-wave is a routine measure of photoreceptor health in the clinic and in animal models of retinal disease^62–64^. In dark-adapted animals, light stimuli produced reduced b-wave amplitudes in *cfap20*^−/−^ homozygotes at both 1.5 mpf and 4 mpf (~50% and 65% reduction; *P* = 0.0382, 0.0017 respectively; Fig. 7e). Conversely, in light-adapted tests, no significant difference in b-wave amplitude was detected at 1.5 mpf, but there was significant reduction observed at 4 mpf (~10% and 65% reduction; *P* = 0.6010, <0.0001 respectively). These data are consistent with retinitis pigmentosa disease progression, whereby rod degeneration precedes cone photoreceptor dystrophy.

IRD typically includes progressive neurodegeneration of the photoreceptor layer. Therefore, we assessed photoreceptor integrity in *cfap20*^−/−^ homozygotes at various ages, using immunohistochemistry to characterize rods and cones (using antibodies 4C12 and Zpr-1, respectively). Rods and cones displayed progressive disorganisation and dysmorphia between 1.5 and 8 mpf, and photoreceptors appeared shorter than age-matched controls (Fig. 7f). Large portions of the cone cell outer segment were detached at 4 and 8 mpf (Fig. 7f arrowheads) and may undergo phagocytosis by the retinal pigment epithelium. TEM imaging revealed unwinding of the OS membranous discs (Supplementary Fig. 12) and engulfment of detached outer segments by the RPE (Fig. 7g). These phenotypes were not observed in WTs controls or in mutants during larval stages (Supplementary Fig. 10h). Furthermore, activated caspase-3 positive cells were detected in the photoreceptor layer of cfap20^*−*^^/−^ homozygotes at both 4 and 8 mpf, indicative of apoptotic cell death, and the density of nuclei in the ONL was reduced in mutants (Fig. 7h). Taken together, these data suggest that *cfap20*^−/−^ homozygotes succumb to progressive retinal dystrophy with mild developmental defects and severe histological changes in adulthood.

## Discussion

**Fig. 8:**
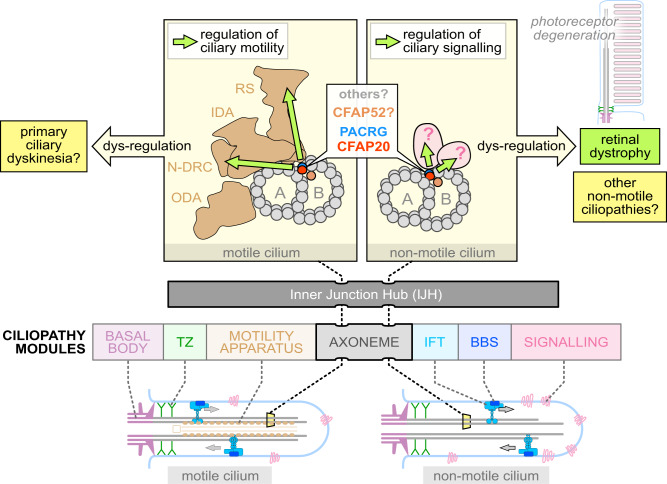
Model depicting the Inner Junction Hub (IJH) as regulatory domain in motile cilia and previously uncharacterised ciliopathy (retinal dystrophy) region that influences the signalling functions of non-motile (primary) cilia. Ciliopathies most commonly arise from the dysfunction of well-characterised functional modules (ciliopathy modules), namely the basal body, the transition zone (TZ) ciliary gate, intraflagellar transport (IFT) or IFT-associated BBS cargo-trafficking system, a variety of signalling proteins, and a motility apparatus specific to motile cilia. Distinct from these macromolecular complexes are proteins within and near the IJ including CFAP20, PACRG and potentially CFAP52, which may represent a previously unknown ciliopathy module that influences ciliary beat patterns (motility) likely by acting via radial spokes (RS) and the Nexin-Dynein Regulatory Complex (N-DRC). Our study confirms a role for zebrafish CFAP20 in metazoan motile cilia, implicating it as a strong candidate for the motile ciliopathy, primary ciliary dyskinesia (PCD). In *C. elegans*, CFAP20 and PACRG independently regulate the signalling functions of different classes of non-motile cilia. Lastly, mutations in human CFAP20 result in retinal dystrophy, a ciliary photoreceptor degeneration phenotype that is phenocopied in zebrafish CFAP20 mutant animals. In these non-motile cilia, the IJH may influence, structurally and/or functionally, currently unknown signalling proteins, and represents a previously unknown disease locus for photoreceptor degeneration. IDA Inner Dynein Arm, ODA Outer Dynein Arm, BBS Bardet-Biedl syndrome. Shapes and symbols in this schematic are also represented in the complementary Fig. 1.

Ciliopathies represent a prevalent, diverse spectrum of diseases that show complexity in mechanism and the organs impacted. A growing list of ~200 genetic loci underpin cilium-associated human disorders, virtually all of which are categorised as either motile, or non-motile, ciliopathies^12,13^ (Fig. 1a). Here, we attribute a rare form of recessive inherited retinal dystrophy (IRD; a classic non-motile ciliopathy^10,65,66^) to variants in *CFAP20*, which encodes a remarkably conserved protein with well-established roles in motile cilia from diverse organisms^15–18^. Our studies using zebrafish, *C. elegans*, and mammalian cell culture model systems collectively demonstrate functions for CFAP20 in both motile and non-motile metazoan cilia. The findings support a model (Fig. 8) whereby CFAP20 plays a critical role within a structural/functional hub centered in and around the inner junction of ciliary double microtubules (Inner Junction Hub or IJH), which likely regulates both motile cilia beating and non-motile cilia function. Importantly, disruption of the IJH represents a previously uncharacterised ciliary aetiology of IRD, and perhaps more generally, ciliopathies.

Our zebrafish studies identify a critical function for CFAP20 in tissues bearing motile cilia. Consistent with previous transient knockdown (morpholino) studies^16^ we demonstrate that *cfap20* mutants show classical motile ciliopathy phenotypes, including severe body axis curvature defects, cardiac situs defects, and pronephric duct cysts^27,67–70^. Adult homozygous zebrafish were also infertile, but we cannot rule out that this was due to poor fitness. Intriguingly, one family of *CFAP20* patients with syndromic IRD and fertility issues were identified. Combined with sperm motility defects in *Drosophila cfap20* mutants^23^, our findings suggest that CFAP20 might be generally required for regulating motile cilia function in diverse animals—as it does in the unicellular organisms *Paramecium* and *Chlamydomonas*^15–18^.

The human *CFAP20* genotypes we identified thus far are all biallelic missense or missense combined with a canonical splice site variant (i.e., likely to be non-null genotypes). Taken together with the low survival rate of zebrafish *cfap20*^−/−^ mutants, this may hint that *CFAP20* is an essential gene in humans, and that its pathogenic spectrum might be constrained. Indeed, the evolution of CFAP20 is itself highly limited since its protein sequence has remained remarkably conserved since the dawn of eukaryotes (Supplementary Fig. 1). The multiple contacts of CFAP20 with ciliary axoneme tubulins and PACRG, as well as proximity to CFAP52 and other proteins at or near the inner junction^19–21,71^ (Figs. 1b and 8), could explain this degree of conservation. Our experimental findings also support the notion that the patient missense variants in *CFAP20* (p.(Tyr86Cys), p.(Arg102His), p.(Arg113Trp) and p.(Arg153Gly)) may not be null. First, GFP-tagged*C. elegans* CFAP20 proteins bearing the mutations are, similar to wild-type, strongly enriched within the proximal (inner junction-containing) region of the ciliary axoneme (albeit with some signs of mislocalisation; Fig. 6a). Second, the human variants had capacity to rescue the curled tail phenotype of *cfap20*^−/−^ homozygote zebrafish (except p.(Tyr86Cys)) when overexpressed (Fig. 6b), indicating retained functionality to rescue this particular motile cilium-associated developmental process. The variant that failed to rescue—p.(Tyr86Cys)—was also associated with a syndromic IRD, consistent with this mutation being the most damaging to function, in the context of motile cilia at least.

Another key finding from our study is the requirement for CFAP20 in maintaining the function and structural integrity of zebrafish photoreceptor outer segments (i.e., specialised cilia). Photoreceptor development was grossly normal in mutants at seven days of age, but since *cfap20* is maternally inherited (Supplementary Fig. 10) we cannot rule-out that this obscured a requirement for *cfap20* in early retinal development. Clear morphological disruption of the retina and ERG anomalies are apparent at 1.5 months post fertilisation (mpf), with further progression at 4 and 8 mpf. This degeneration phenotype observed in adult fish is consistent with the retinal dystrophy diagnosed in *CFAP20* patients. While our study was in revision, González-del Pozo et al.^72^ identified *CFAP20* as a putative cause of autosomal recessive RP from a cohort of 14 whole genome sequences from genetically undiagnosed IRD patients; this study identified one individual with homozygous p.(Arg113Trp) *CFAP20* variants that segregated with disease. This additional study independently strengthens the case for *CFAP20* mutations being associated with IRD. Retinal dystrophy represents a common clinical phenotype in several non-motile ciliopathies^12,73,74^. For example, defects in proteins associated with intraflagellar transport (*e.g*., IFT140, TULP1), Bardet-Biedl syndrome (*e.g*., BBS1, BBS2, ARL6), the transition zone ciliary gate (*e.g*., CEP290, RPGR), and photoreceptor-specific or general ciliary signalling proteins (ARL3, CNGA1, etc.) all include retinal dystrophy amongst their presentations^12^. How CFAP20 functions to prevent photoreceptor cilium degeneration is unclear but could include regulating axoneme length and/or axoneme stability for example by post-translational modification^21,23^, or impaired transport or signalling, as mentioned above. The role for CFAP20 in photoreceptors, which have elaborate light-gathering outer segments based upon canonical ciliary structures (axonemes), also hints at the possibility of a more general role for CFAP20 in non-motile cilia, a notion supported by its presence not only in photoreceptor but also primary cilia proteomes^18,53^.

The importance of CFAP20 in non-motile cilia is also illustrated by its existence in *C. elegans*. Having secondarily lost motile cilia during evolution, nematode genomes have purged virtually all genes associated with cilium motility. Yet, *C. elegans* CFAP-20 participates in several sensory and physiological processes that depend on various types of non-motile cilia: ability to sense and learn to avoid salt in the absence of food (gustatory plasticity); lifespan control; locomotion (roaming); and, body size determination^40,42,47–51^. While *C. elegans cfap-20* mutant cilia are superficially intact when observed by fluorescence microscopy and exhibit correct trafficking of the intraflagellar transport (IFT) system (Supplementary Fig. 5c, 8b), at least one class of cilium is elongated (Fig. 3b), similar to CFAP20-depleted mammalian primary cilia (Supplementary Fig. 6 and ref. 23). Why some cilia are longer is unclear but could, for example, relate to subtle changes in IFT, which regulates ciliary length, or differences in post-translational modifications (axoneme stability/stiffness). More strikingly, TEM ultrastructural analysis reveals a specific lesion at the axonemal inner junction. In most cilia examined, the B tubule fails to close a seam that completes the doublet microtubules (Fig. 3c and Supplementary Fig. 7). This is different from *Chlamydomonas*, where loss of CFAP20 on its own causes the loss of an internal beak structure but does not result in obvious structural damage to the doublet^16^. We surmise that the disrupted inner junction in *C. elegans* non-motile cilia may disrupt physical/functional associations with currently unknown signalling protein(s) within this axonemal region, and thus result in the observed sensory/signalling defects. Alternatively, loss of CFAP20 (or the PACRG inner junction protein) could destabilise the axoneme structure (including the inner junction) of non-motile cilia, which might indirectly affect signalling functions.

Notably, disruption of the *C. elegans* PACRG orthologue engenders essentially the same array of behavioral and developmental phenotypes observed upon deleting CFAP20 (Fig. 4). In *Chlamydomonas*, loss of either protein (CFAP20/PACRG) results in a similar dysregulation of ciliary motility^16,17,75^. The two proteins physically interact and constitute the alternating pair of proteins that makes up the inner junction (Fig. 1). In both *C. elegans* and *Chlamydomonas*, CFAP20 and PACRG localisation to this region is independent from each other (Fig. S5b; ref. 20). Together, these data provide complementary evidence that proteins within the inner junction have comparable, non-redundant regulatory roles in supporting the functions of either motile and/or non-motile cilia.

Aside from helping to form a bridge between A and B tubules, what specific functions CFAP20 might have in motile or non-motile cilia are not understood. From cryo-EM structures of *Chlamydomonas* and *Tetrahymena* motile cilia, proteins at or near the inner junction, which include CFAP20, PACRG, CFAP45, CFAP52, CFAP90, CFAP126 and EFHC1^19–21^, are all positioned to form a potential scaffold for, or functionally influence, the radial spokes and dynein regulatory complex that regulates ciliary motility (Fig. 8). Consistent with such a role, in *Chlamydomonas* or other unicellular eukaryotes, CFAP20, PACRG, CFAP45, CFAP52 and EFHC1/Rib72 are all needed to regulate ciliary beat patterns^20,76–78^.

Interestingly, in humans, CFAP45 and CFAP52 are both associated with a human laterality defect but not PCD^79,80^. While not conclusive, these findings suggest a potential role for these proteins in the non-motile cilia of the embryonic left-right organiser, which confer laterality by sensing the directional flow of fluid created by motile cilia. At the same time, mutations in CFAP45 cause reduced sperm motility, suggesting a defect in motile cilia^79^. Another protein, Enkurin (CFAP106) also localises near the inner junction and its disruption similarly leads to *situs inversus* in humans^81^, with ciliary motility defects also as a potential aetiology^82^. The protein tektin is also likely associated with the inner junction, as its ciliary presence is reduced in the *Chlamydomonas* CFAP20 mutant^16^. Notably, a paralogue (TEKT1) in humans represents a motile ciliopathy candidate^83^. EFHC1 is associated with ciliary signalling in both human cells and *C. elegans*, but its disruption in humans results in juvenile myoclonic epilepsy, whose aetiology could potentially result from non-motile cilia dysfunction^84,85^. Remarkably, there is even a discontinuity in the CFAP20-PACRG arrangement at the inner junction, where a periodic absence of PACRG coincides with the association of the N-DRC (Nexin-Dynein Regulatory Complex) with the axoneme^19,20^. Recent reports link PACRG to human sperm dysfunction (Astheno-teratozoospermia)^86^, laterality defects in frog^87^, and a mouse knockout displays ciliary motility defects and hydrocephalus^88^.

Our findings, coupled with published observations, provide strong evidence that disruption of inner junction-associated proteins often influence the functions of motile cilia, but may have unexpected roles in non-motile cilia as well. As such, the inner junction proteins may be associated with either motile and/or non-motile ciliopathies.

Ciliopathies can manifest as a non-syndromic disease involving a single organ system, or a pleiotropic disorder that affects multiple organ systems^89–91^. We present 8 individuals formally diagnosed with retinal dystrophy, a clinical phenotype linked to both non-syndromic and syndromic ciliopathies (Fig. 5a, Table 1). We propose that human CFAP20 may function not only in photoreceptor cilia, but also in other cell types bearing non-motile cilia. It is not yet well understood why disruption of some ubiquitous ciliary proteins often only manifest as a retinal dystrophy. However, we hypothesise that the genotypes observed in the affected individuals with CFAP20-related IRD are non-null, and that more severe *CFAP20*-associated disease may occur with more damaging genotypes, or be incompatible with life. Potentially consistent with this notion, genomic analysis as part of the 100KGP^92^ revealed biallelic rare protein-altering variants affecting CFAP20 in only 4/70,000 individuals. Three of these individuals were recruited to the study with a retinal dystrophy (F1-F3) and independently a pedigree in Canada was identified (F4) with a p.(Tyr86Cys) variant. Given the absence of any other convincing molecular diagnosis or candidate variants to explain the fertility and neurological features in family F4, we speculate that these cases may represent a syndromic manifestation of CFAP20-associated disease. Additional families will need to be identified to clarify the phenotypic spectrum of CFAP20-related disease, as different variants may be associated with differential (i.e., less or more severe) functional impairment of the protein.

In conclusion, our work challenges the prevailing view that functional defects in either motile or non-motile cilia stem from mutations in distinct groups of proteins and cause clinically separable disorders^12,13^. We suggest that a number of different axoneme-associated proteins—including CFAP20, PACRG and others at or near the inner junction—should be investigated not only for potential roles in human cilium motility and involvement in PCD, but also for associations with non-motile cilium signalling and ciliopathies, including but not restricted to retinitis pigmentosa.

## Methods

### Zebrafish strains and protocols

#### Zebrafish lines and husbandry

Fish were raised at 28.5 °C under a 14/10 h light/dark cycle. Anaesthesia was obtained by 164 mg/L Tricaine. All fish were on AB background and staged as in Kimmel et al.^93^. Transgenic lines used are detailed in Supplementary Table 4: Tg(−5.5opn1sw1:EGFP)kj9^94^, Tg(−3.7rho:EGFP)kj2^95^. Embryos were raised at 28.5 °C in E3 media^96^. All experiments were performed with at least 10 larval (<5dpf) zebrafish per condition and at least 3 adult (1.5 mpf, 4 mpf, and 8 mpf) zebrafish. Sex determination was not possible due to lack obvious sexual dimorphism in *cfap20*^-/-^ homozygotes.

#### Morpholino oligonucleotide and mRNA overexpression

Splice morpholino oligonucleotides (MO) (Gene Tools LLC) as in Yanagisawa et al.^16^, were injected into the yolk of 1 cell stage (CS) embryos at a final dose of 1, 2 or 4 ng (2 clutches, >30 animals). Treatment group phenotypes were scored and quantified at 48 h post fertilisation (hpf), and cardiac looping situs was recorded (Fig. S3, S4). An optimal dose of 3 ng *cfap20* MO was used in subsequent experiments. For rescue experiments, mRNA was transcribed using mMessage mMachine kit (ThermoFisher AM1340) and phenol:chloroform purified. mRNA was serially diluted in ddH_2_O to the desired concentration and injected into the cell at the 1 or 2 CS. Each of the conditions (i.e. mCherry, WT *CFAP20*, variant *CFAP20*) were injected into embryos from the same clutch, in ≥3 clutches total. Unfertilised or embryos stalled during gastrulation were removed at 12 hpf. At 48 hpf the clutches were blinded and scored for body axis curvature. Embryos were then processed for DNA lysis and genotyped as below. Following genotyping the embryos were unblinded and only *cfap20*^-/-^ homozygote data was analysed. The absolute number of norma’ vs axis defects was compared statistically between WT *CFAP20* and variant *CFAP20* injected using Fisher’s exact test.

#### Generation and genotyping of zebrafish cfap20 mutant allele ua5025

CRISPR/Cas9 mutagenesis was performed as in Gagnon et al.^97^. Briefly, the CHOPCHOP server was utilised to identify PAM sequences in *cfap20* neighbouring the orthologous region of the c.257A > G mutation in family 4^98^. CGAATGTGAAATACTTCTTGand GGAAAGGAAGCTTGATGCCG sequences were selected to target exon 3 and two targeting primers with SP6 promoters and a constant oligo were ordered from Integrated DNA Technologies. Oligos were hybridised and elongated using T4 DNA polymerase (NEB M0203S) to generate a template for transcription. The SP6 MEGAscript kit (Thermofisher AM1330) was used to generate the two gRNAs which were then 1:1 mixed and injected at 600 ng/µl with 0.75 µL EnGen Spy Cas9 NLS protein (NEB M0646M) into the one-cell stage zebrafish embryos. Mosaic F0 embryos were raised to 3 months and outcrossed to generate F1 *cfap20*^+/−^ heterozygote animals. Approximately 10 embryos per clutch were collected for gDNA extraction^99^ and PCR amplification surrounding the cut sites, followed by ligation into pGEM-T (Promega A3610) was performed followed by Sanger sequencing to identify mutations. Clutches with the ua5025 allele were raised to adulthood and outcrossed to generate an F2 generation. All experiments were performed on progeny of the F2 generation. PCR genotyping of ua5025 individuals was performed using flanking primers: Fwd—GCACCACATACATCACGTGTCC, Rev—AGAAAAGCTCCAAGAGAAACACACA. The 7 bp difference was resolved by SB buffer agarose electrophoresis^100^.

#### Cardiac situs and gut situs scoring

Cardiac scoring was performed at 28 hpf (jogging) and 48 hpf (looping) by brightfield microscopy (using QCapture 5) in live embryos. Scoring was based on^101^. Gut situs was scored following in situ hybridisation of 48hpf embryos with *foxa3* probe^102^.

#### In situ hybridisation

Wholemount in situ hybridisation was performed essentially as described in^103^. Briefly, digoxigenin-labelled probes were synthesised from linear DNA templates using SP6 RNA polymerase (Sigma 10810274001) and digoxigenin-UTP labelling mix (Sigma 11277073910) and purified via TRIzol/chloroform extraction (Thermofisher 15596026) and ethanol precipitation. Fixed embryos were ermeabilized with 1 µg/ml proteinase K treatment for 15 mins (48 hpf), re-fixed with 4% PFA for 20 min, and hybridized with 1:200 RNA probes overnight at 65°C. High stringency washes in 0.2x and 0.1 x SSC/0.1% Tween 20 were carried out at 65^o^C for 20 minutes, and blocking was performed for 1 h in 2% sheep serum, 2 mg/ml bovine serum albumin. Anti-digoxigenin-AP Fab fragments (Sigma 11093274910) at 1:5000 dilution was used to detect probe hybridisation, and NBT/BCIP (Sigma 11681451001) colouration reactions were performed at 38 °C until signal was saturated. The probes used were *myl7*^104^ and *foxa3*^102^.

#### Retinal histology

For resin sections: tissue from dark adapted animals was fixed in 4% paraformaldehyde (PFA) overnight at 4 °C and transferred by gradient up to 100% ethanol. Tissue was embedded in Technovit® 7100 resin (Technovit 64709003) and cured in a dehumidified environment for at least 1 week. 5 µm sections were produced and stained with Methylene blue/basic fuchsin as in^105^. Sections were mounted with Permount™ Mounting Medium (Fisher Scientific SP15-100) and imaged. Globe diameter was measured in FIJI software v.1.8.0^106^ on sections containing the optic nerve. In larval zebrafish, photoreceptor transgenic lines were used to examine photoreceptor subtypes. At 7 dpf larvae were fixed in PFA and bleached for 20 min with 0.5% KOH and 3% H_2_0_2_. Samples were blocked in 5 mg/ml bovine serum albumin, 5% donkey serum, and 1:2000 dilution of rabbit anti-GFP primary antibody (Invitrogen A-11122) was used in block overnight. Secondary 1:2000 488 Alexafluor® goat anti-rabbit (ThermoFisher Scientific A-11008) and 1:2000 nuclei stain TO-PRO™−3 Iodide (ThermoFisher Scientific T3605) in block was performed for 2 h. Samples were deyolked with fine forceps and flatmounted in 70% glycerol (ThermoFisher Scientific 15514011). 1μm Z-interval confocal stacks were taken on a LSM 700 confocal microscope (Zeiss, running Zeiss Zen 3.2 software) with 40x oil lens and 5-7 images used for maximum intensity projection. FIJI was used to record the length of ~10 photoreceptors per animal from synapse to OS tip.

For adult tissues, dark-adapted enucleated eyes with lenses removed were fixed, cryoprotected in 35% sucrose, mounted in Tissue-Tek® O.C.T. (Sakura, 4583), and cryosectioned at 10 µm thickness. Tissue on slides was bleached and immunostained as above using 1:200 Zpr-1 (ZIRC ZDB-ATB-081002-43), 1:200 4C12 (ZDB-ATB-090506-2 a kind gift from James Fadool), or 1:200 cleaved caspase-3 (Cell Signalling Technology #9661) in block. Secondary antibodies 1:200 555 Alexafluor ® donkey anti-mouse or 488 Alexafluor ® chicken anti-rabbit (ThermoFisher Scientific A-31570, A-21441) and 1:500 nuclei stain TO-PRO™−3 Iodide in block were incubated on samples for 2 h. Sections were mounted with ProLong™ Gold Antifade Mountant (ThermoFisher Scientific P36930) and confocal imaged with 5 Z-stacks used for maximum intensity projection.

Transmission electron microscopy (TEM) was performed as in^107^. Briefly, enucleated eyes with lenses removed were fixed in 2.5% glutaraldehyde/2% paraformaldehyde for 3 days, halved, washed with 0.1 M phosphate buffer, and osmium tetroxide treated. Tissue was gradually dehydrated in ethanol and embedded in Spurr resin with overnight curing at 70 ^o^C. Ultrathin (80 nm) sections were transferred to formvar grids (Millipore Sigma TEM-FCF400CU50) and uranyl acetate/lead citrate post stained. Grids were imaged using a Morgagni 268 (Philips/FEI) at 80 mV.

#### Zebrafish optical coherence tomography and electroretinogram

Optical coherence tomography on zebrafish was performed as previously described^107^. In brief, zebrafish were anesthetised in tricaine solution and placed on a triangular sponge inside of a plastic chamber, right eye facing upward. A weighted strip of gauze was placed across the fish’s body to prevent movement and the subject was covered in tricaine solution. A handheld Envisu R-Series OCT (Bioptigen Inc., Durham, NC, USA) was mounted perpendicularly to the zebrafish and InVivoVue 2.4 OCT Management Software (Bioptigen Inc., Durham, NC, USA) used for acquisition and analysis. Once the procedure was completed, the animals were placed in a recovery tank. ERGs were performed as in Nadolski et al.^108^. Briefly, animals were dark adapted (where necessary) for 30 min and anaesthetised for 2 min until motionless. Animals were transferred to a damp moistened polyvinyl alcohol sponge above the reference electrode and moved into a Ganzfeld light stimulator connected to an E3 Electrophysiology System (Diagnosys LLC, Lowell, MA, USA). An Ag/AgCl recording electrode was positioned on the cornea and a testing protocol of five dim (dark adapted–3 cd.s/m^2^) or bright (light adapted–10 cd.s/m^2^) white light stimuli flashes separated by 5 s was recorded and averaged. A band-pass filter of 0.3–300 Hz was applied to reduce system noise.

### Micro-computed tomography

Tissue samples were processes as in ref. 109. Briefly, *n* = 3 sacrificed, enucleated WT and *cfap20*^−/−^ zebrafish were fixed in 3 ml 4% PFA, shaking for 48 hpf. Tissues were scanned using MILabs μCT using the parameters 50 kV voltage, 0.24 mA current, 75 ms exposure time. Scans werre exposed to cycloheximidee reconstructed with 3D Slicer software^110^ and images exported as Tif format.

### 5′ RACE, RT-PCR, and qPCR

Thirty *cfap20*^−/−^ homozygotes and AB controls at 72 hpf were collected and used for RNA extraction using TRIzol reagent (Thermofisher Scientific 15596026) as per the manufacturer’s instruction. RNA quality >RIN 8 was ensured on 2100 Bioanalyzer (Agilent) and then SMARTer® 5′ RACE (Takara Bio 634858) was performed using a *cfap20* specific reverse primer GATTACGCCAAGCTTCGAGAAGCGTGGAGA AACCCTGCCG. The positive and negative controls provided in the kit were also performed. The 5′ RACE products were resolved on a 1% agarose/TAE gel and the major bands at ~700 bp were subcloned into pGEM-T (Promega A3600) and sanger sequenced to confirm that they were the known isoform of *cfap20*.

For RT-PCR, pooled embryos of the desired stage underwent RNA extraction as above before being used in reverse transcription reactions (Thermofisher 4368814). Cfap20 and β-actin specific primers were used in the PCR at 33 and 25 reactions respectively (Promega M7848). PCR products were resolved on a 1% agarose/TAE gel. Each timepoint was repeated with 3 biological replicates (different clutches). The qRT-PCR was performed as in^111^. Briefly, RNA was collected at 72 hpf and cDNA reverse transcribed as above. cDNA was diluted 1:10 and validated using a standard serial dilution to determine efficiency under MIQE guidelines^112^. The proprietary Dynamite qPCR master mix (MBSU, UofA) was used for all reactions with cfap20, and gapdh/β-actin primers (Supplementary Table 4) as housekeeping controls. Two-step amplification was performed on the 7500 Fast Real-Time PCR System (Applied Biosystems) and fold change calculated using the delta-delta Ct method, normalised against β-actin. Three technical replicates were performed per experiment, with four biological replicates per sample.

### Statistical analyses and image manipulation

All graphing and statistical tests regarding zebrafish data were produced in GraphPad Prism 9. Discrete data was analysed via Fisher’s Exact test. Normal continuous data was analysed via unpaired T-test or one-way ANOVA and Dunnett’s post-hoc test. Data that failed a Shapiro Wilk test were analysed via unpaired Mann-Whitney test. Raw images were cropped and brightness/contrast was adjusted in Photoshop V. 22.4.3. Identical transformations were performed on control vs experimental condition images.

### C. elegans strains and protocols

#### C. elegans strains used and constructed

Wild-type and mutant strains (Supplementary Table 1) used in this work were obtained from the *Caenorhabditis* Genetics Center (CGC) (https://cgc.umn.edu/), except FX2597 (National Bioresource Project shigen.lab.nig.ac.jp/c.elegans/index.jsp) and PHX627 (ordered from SunyBiotech; www.sunybiotech.com). The *cfap-20(syb627)* null mutant was generated by CRISPR-Cas9 and deletes 1453 bp of *cfap-20*/C54C6.6 (flanking sequences CACGTTCCAA—TGAAGGATGG), leading to a frameshift/stop after 7 amino acids. Wild-type *C. elegans* Bristol N2 or PD1074 strains were used^113,114^. Translational GFP reporter constructs (CFAP-20::GFP & PCRG-1::GFP) were generated following a protocol similar to previously published^115,116^. GFP-tagged CFAP20 constructs bearing patient mutations (p.(Tyr86Cys), p.(Arg102His), p.(Arg113Trp) and p.(Arg153Gly)) were created by PCR mutagenesis and confirmed by sequencing. All strains were cultured and maintained at 20 °C unless noted otherwise^113^.

#### Salt chemotaxis assays

Salt chemotaxis assays were performed as previously reported^34,117^. Briefly, washing worms off the plates with low salt CTX buffer, sedimentation for 5 min., removal of supernatant and placing a drop containing 100 to 200 worms in the center of a plate with two 1 mM salt and two 0 mM NaCl containing agar quadrants. Liquid was wicked away with wipes and plates scored after 10 min. The chemotaxis index for each plate and time point was calculated as (A—C)/(A+C). A being equal the number of animals on the quadrants with NaCl, and C is the number of animals localizing on the quadrants with 0 mM NaCl. Animals were tested with 0 mM and 1 mM NaCl (salt)-containing quadrants. The behavior of mutant strains was always compared to experiments with control strains on the same day. Each experiment was conducted with at least 4 replicates for each strain.

#### Gustatory plasticity assays

Testing for gustatory plasticity involves pre-exposure to a solution containing 100 mM or 0 mM NaCl for 15 min. and was assessed as described^117,118^. The chemotaxis index for each experiment was calculated as above for salt sensing assays, on quadrant plates probing quadrant plates with 0 and 25 mM NaCl concentration. Worms were cultured at 25 °C.

#### Lifespan assays

Lifespan assays were conducted following a previously published protocol^43^. Worm strains were synchronized by growing until adult stage and eggs harvested via Sodium Hypochlorite lysis. Eggs were transferred for hatching on NGM plates (supplemented with Nystatin, Streptomycin) covered by a lawn of *E. coli* OP50-1. At L4 stage, 100 worms for each strain were transferred to fresh plates (20 worms/plate), a procedure repeated every two days until reaching menopause. Worms were observed for showing signs of life every 1–2 days and alive/dead animals counted. Individuals with exploded vulva, bags or unaccounted worms (having left the plate) were censored.

#### Roaming assays

Roaming assays were carried out as reported^119^ Synchronized L4 stage worms were transferred to NGM plates covered entirely by an OP50 lawn. One worm per plate. After 24 h, worms were picked off and the plate scanned for tracks in the lawn. Using a grid dividing the plate into ~500 squares, each square touched by a track was scored, before calculating the average roaming score from all plates per strain.

#### Body size measurements

To measure body lengths, approximately 50 gravid adult worms from each strain were transferred to a fresh NGM plate seeded with OP50 and left to lay eggs and removed after 2 h. The resulting progeny was left to grow at 20 C for a further 70 h and imaged (using Volocity version 6.5.1). The length of 50 worms for each strain was measured with the segmented line tool in ImageJ v.1.53e^120^.

#### General imaging

Worms were immobilized with 40 mM levamisole or 0.1 μm diameter polystyrene microspheres in water (Polysciences 00876‐15, 2.5% w/v suspension) on 10% agarose inwater pads. Unless noted otherwise, imaging was performed on a spinning disc confocal system (WaveFX system from Quorum Technologies), consisting of an inverted Zeiss AxioObserver equipped with a 100× oil (N.A. 1.4) objective, Yokogawa CSU-10 spinning disk head, 491 & 561 nm lasers, and a Hamamatsu C9100‐13 EMCCD camera, controlled by Volocity software version 6.5.1 (PerkinElmer). Body-length imaging was completed on a Zeiss Axioskop 2 mot with a 100× oil (N.A. 1.3) objective, FluoArc/HBO103 illuminator, via a Hamamatsu Orca ER C4742‐80 CCD camera and Open Lab (V5) (Agilent) software.

#### Intraflagellar transport velocity measurements

IFT speeds were assayed similarly to that described by Jensen et al.^121^ Time-lapse movies of OSM-3::GFP and CHE-11::GFP reporters expressed in the *cfap-20(syb627)* mutant were loaded into ImageJ and Kymographs prepared with the KymographClear plugin^122^ version 1.0. Subsequently, tracks were assessed with the ‘straight line’ tool of ImageJ^120^ and the line angles employed to calculate IFT speeds.

#### Cilia length measurements

The segmented line tool of ImageJ^120^ version 1.53e was employed to measure the length of cilia in confocal images of OSM-3::GFP and CHE-11::GFP transgenic worms.

#### Transmission electron microscopy

Transmission electron microscopy analyses of *C. elegans* head cilia, which involved fixing, sectioning and imaging, were performed on young adult worms as previously reported^119^. Imaging of one worm for each strain was performed on a FEI Tecnai T12.

### Statistical analyses

Unless noted otherwise, statistics to evaluate significance of results was performed with Graphpad Prism version 5.0.1. Unless indicated, experimental data passed a Shapiro-Wilk test, *P* values were determined with one-way ANOVA and Tukey-Mann post-hoc test. Otherwise, a Kruskal-Wallis test and Dunn’s multiple comparisons test were employed to determine significance. Lifespan assays were quantified by Kaplan-Meier survival analysis. The gustatory plasticity assays were analysed in SPSS version 25 employing one way ANOVA and Bonferroni post hoc test. Whiskers in box plots show min/max values.

Unless noted differently, all experiments were repeated at least twice on different days, each with an independently grown worm cohort. Individual worms were used for one experiment, before being discarded. Worms with infections (burst vulva phenotype) were excluded from experiments.

All *C. elegans* strains, materials, and data are available from leroux@sfu.ca on request.

### Affected individuals and mutational analyses

#### Clinical investigations

Family 4 were assessed by a medical geneticist (PYBA) and all families assessed by an ophthalmologist (F1: ARW, F2: FD, F3: SD, F4: IMM). Affected individuals underwent clinical ophthalmic examination including (where possible) best-corrected Snellen visual acuity (VA), dilated fundus examination, colour fundus photography (Optos), fundus autofluorescence (FAF), spectral domain ocular coherence tomography (SD-OCT). Multifocal electroretinography (mfERG) was recorded with the Espion system (Diagnosys, Lowell MA USA) using DTL electrodes according to ISCEV standards (www.ISCEV.org). Full field ERGs (ffERG) were recorded with the UTAS system (LKC, Gaithersburg, MD USA).

#### Genetic analyses

Affected individuals F1:1, F1:2, and F1:3 underwent autozygosity mapping with the Genome-Wide SNP 6.0 array (Affymetrix, Santa Clara, CA, USA) to identify shared autozygous chromosomal segments as previously described^123^. Subsequent exome sequencing was performed (proband only) and rare (MAF ≤ 0.005) homozygous protein-altering variants were investigated within the shared autozygous regions as previously described^124^. Individuals F1:1, F2:1, and F3:1 underwent genome sequencing as part of the UK100KGP, as previously described^125^. The index cases underwent the full clinical variant interpretation pipeline to identify and interpret pathogenic or likely pathogenic variants in the retinal dystrophy gene panel. (https://panelapp.genomicsengland.co.uk/panels/307/) including variant interpretation according to the ACGS clinical guidelines^126^.

Following negative gene panel analysis, the families were analysed as part of the unsolved retinal dystrophy cohort of individuals in the 100KGP: Rare, protein altering, possible biallelic variants (MAF ≤ 0.001 in the 100KGP cohort and the gnomAD dataset) were identified across the entire genome. For individual F1:1, the analysis was focused on homozygous variants in the previously identified shared regions of autozygosity. Subsequently, for individuals F2:1 and F3:1, potential biallelic genotypes across all genes were investigated.

Family 4 underwent clinical whole exome sequencing through Blueprint Genetics (parents and all three sibs were sequenced). This family also had research-based exome sequencing through the Mito-Find protocol as described by Kerr et al.^127^, which did not identify any other rare candidate variants associated with retinal disease or the other neurological symptoms in this family.

Variants were all confirmed by PCR and direct Sanger sequencing where possible including family segregation analysis (Fig. 5a). PCR and direct Sanger sequencing of all coding exons of *CFAP20* including splice junctions was performed on a cohort of 200 unsolved retinitis pigmentosa patients ascertained from the Inherited Eye Diseases clinic at Moorfields Eye Hospital (primers and conditions available on request).

### Mammalian cultured cell protocols

#### Protein half-life assays

Protein half-life for each of the discovered variants (p.(Tyr86Cys), p.(Arg102His), p.(Arg113Trp), p.(Arg153Gly)) were carried out using exogenously expressed CFAP20 tagged at the C-terminal with a Myc tag (pCMVk-CFAP20 -Myc). 2.5 ug of vector was transfected into HEK293T cells (ATCC® CRL-11268) with Lipofectamine 2000 (Thermofisher, USA) as per the manufacturer’s instructions. Transfected cells were exposed to cycloheximide (CHX) dissolved in 95% ethanol at 50ug/uL for 0, 1, 2, 3, 4, 5, and 10 h. Cells were harvested in Radioimmunoprecipitation (RIPA) lysis Buffer (Thermofisher Scientific, USA) and 50 ug of protein was loaded onto polyacrylamide gels and ran at 100 V for 2 h. Polyacrylamide gels were transferred to nitrocellulose membranes at 100 V for 1 h and blocked with Intercept (PBS) Blocking Buffer (LI-COR Biosciences, NE, USA) for an hour or overnight. Blots were probed with antibodies for the Myc tag (1:1000 dilution, Cell Signalling Technology, MA, USA) and for a loading control, β-actin (1:12,000 dilution, Santa Cruz Biotechnology, CA, USA). Blots were then probed with Goat Anti-Mouse IgG Polyclonal Antibody (1:12,000 dilution, IRDye® 800CW), and Goat Anti-Rabbit IgG Polyclonal Antibody (1:120,00 dilution, IRDye® 680RD) (LI-COR Biosciences NE, USA) for 1 h at 4 degrees. Blots were visualized and bands quantified using the iBright Western Blot Imaging system (ThermoFisher Scientific, MA, USA). Band intensities were normalized to the loading control and half-life of each protein variant was calculated using the formula for first-order decay kinetics (Ln(0.5)-(y-intercept/slope)). Half-life data was assessed for statistical significance using Graphpad Prism. Data were visualized using a simple linear regression analysis and statistical analyses included a QQ-plot to confirm Gaussian distribution of data. Statistical significance was determined via repeated measures one-way ANOVA, and a Dunnett correction for multiple comparisons.

#### Cilia length calculations

To assess effects of CFAP20 on cilia structure, a mouse fibroblast line NIH3T3 was obtained (ATCC® CRL-1658). NIH3T3 cells were plated on glass slides at a density of 300,000 cells/well of a 6-well plate and allowed to grow overnight. Cells were then transfected with a small interfering RNA (siRNA) targeting mouse Cfap20 using Lipofectamine 2000 as per above. Cells were fixed in 2% paraformaldehyde for 20 min, blocked and permeabilised using a Immunofluorescence Blocking Buffer (IFBS: PBS + 1% Bovine Serum Albumin, 0.5% Triton X-100). An antibody for acetylated tubulin (1:500 DILUTION, Millipore Sigma, ON, Canada), a marker for primary cilia, was diluted in IFBS, added to cells and incubated for 1 h at room temperature. Cells were stained with DAPI diluted in PBS (1:10,000 dilution, Millipore Sigma, ON, Canada) to mark nuclei. Donkey anti-Rabbit IgG H + L Alexa Fluor 555 secondary antibody (1:1000 dilution, Invitrogen, MA, USA) was added and glass slides were mounted and visualized with a Quorum Wave FX-X1 Spinning Disk Confocal Microscope. Cilia were counted and measured in FIJI using the line tool. Averages of lengths were taken and expressed as a box-and-whisker plot overlayed on a scatter plot of all technical replicates to illustrate data distributions. Statistical analyses were carried out using GraphPad Prism v9.1.2. Analyses included a QQ-plot to confirm Gaussian distribution of data, followed by an unpaired Welch’s t-test (assumes unequal variance) of siRNA control data vs. siRNA *CFAP20* data.

### Reporting summary

Further information on research design is available in the Nature Research Reporting Summary linked to this article.

## Supplementary information

Supplementary Information

Reporting Summary

## Data Availability

The data for the variants identified in families 1-4 have been submitted to ClinVar under accession numbers SCV002562885 [(CFAP20):c.305G>A (p.Arg102His)], SCV002562886 [(CFAP20):c.257A>G (p.Tyr86Cys)], SCV002562887 [(CFAP20):c.337C>T (p.Arg113Trp)], SCV002562888 [(CFAP20):c.397del (p.Gln133fs)], SCV002562889 [(CFAP20):c.164+1G>A], and SCV002562890 [(CFAP20):c.457A>G (p.Arg153Gly)]. The structure of the axonemal inner junction complex was obtained from the Protein Data Bank (6VE7). C. elegans strains were obtained from Caenorhabditis Genetics Center unless otherwise stated. Zebrafish *cfap20* gene structure information was obtained from ENSEMBL release GRCz11 (ENSDARG00000100514). Data accessibility information for the 100KGP is available online (www.genomicsengland.co.uk/join-a-gecip-domain). All data generated during this study are included in this article, its Supplementary file, and the Source Data file provided with this paper. Source data are provided with this paper.

## Source data

Source Data
