# Supplementary material for: The inner junction protein CFAP20 functions in motile and non-motile cilia and is critical for vision
Source: Nat Commun. 2022 Nov 3;13:6595. doi: 10.1038/s41467-022-33820-w (PMC9633640; doi:10.1038/s41467-022-33820-w)
Supplement: Supplementary file 1 — Supplementary Information [file 41467_2022_33820_MOESM1_ESM.pdf]

Supplementary Information

The inner junction protein CFAP20 functions in motile and non-motile cilia and is critical for vision

Paul W. Chrystal [1,2,*], Nils J. Lambacher [3,4,*], Lance P. Doucette[5], James Bellingham[6], Elena R. Schiff[7,8], Nicole C.L. Noel[2], Chunmei Li[3,4], Sofia Tsiropoulou[8], Geoffrey A. Casey[2], Yi Zhai[5], Nathan J. Nadolski[9], Mohammed H. Majumder[1], Julia Tagoe[10], Fabiana D'Esposito[11,12], Maria Francesca Cordeiro[12], Susan Downes[13], Jill Clayton-Smith[14,15], Jamie Ellingford[14,16], Genomics England Research Consortium[17,], Omar A. Mahroo[6,7], Jennifer C. Hocking[2,9,18,19], Michael E. Cheetham[6], Andrew Webster[7], Gert Jansen[20], Oliver E. Blacque[8], W. Ted Allison[1,2,†] Ping Yee Billie Au[21,†], Ian M. MacDonald[2,5,†], Gavin Arno[6,7,22,†], Michel R. Leroux[3,4,†]

[1] Department of Biological Sciences, University of Alberta, Edmonton AB, Canada
[2] Department of Medical Genetics, University of Alberta, Edmonton AB, Canada
[3] Department of Molecular Biology and Biochemistry, Simon Fraser University, Burnaby BC, Canada
[4] Centre for Cell Biology, Development, and Disease, Simon Fraser University, Burnaby BC, Canada
[5] Department of Ophthalmology & Visual Science, University of Alberta, Edmonton AB, Canada
[6] UCL Institute of Ophthalmology, London, UK
[7] Moorfields Eye Hospital, London, UK
[8] School of Biomolecular and Biomedical Science, Conway Institute, University College Dublin, Belfield, Dublin 4, Ireland
[9] Division of Anatomy, Department of Surgery, University of Alberta, Edmonton AB, Canada
[10] Lethbridge Outreach Genetics Service, Alberta Health Services, Lethbridge, AB, Canada
[11] Western Eye Hospital, Imperial College Healthcare NHS Trust, London, UK
[12] ICORG, Imperial College London, London, UK
[13] Oxford Eye Hospital, Oxford University Hospitals NHS Foundation Trust, Oxford, UK
[14] Manchester Centre for Genomic Medicine, Division of Evolution and Genomic Sciences, School of Biological Sciences, Faculty of Biology, Medicine and Health, University of Manchester, Manchester, UK
[15] Manchester Centre for Genomic Medicine, St Mary's Hospital, Manchester University NHS Foundation Trust, Health Innovation Manchester, Manchester, UK
[16] Division of Evolution and Genomic Sciences, School of Biological Sciences, University of Manchester, Manchester, UK
[17] Genomics England, London, UK; A list of authors and their affiliations appears at the end of the paper
[18] Department of Cell Biology, University of Alberta, Edmonton AB, Canada
[19] Women and Children's Health Research Institute , University of Alberta, Edmonton AB, Canada
[20] Department of Cell Biology, Erasmus University Medical Centre, Rotterdam, the Netherlands
[21] Department of Medical Genetics, Alberta Children's Hospital Research Institute, Cumming School of Medicine, University of Calgary, Calgary AB, Canada
[22] North Thames Genomic Laboratory Hub, Great Ormond Street Hospital for Children NHS Foundation Trust, London, UK
[*] These authors contributed equally
† These authors jointly supervised this work

SUPPLEMENTARY FIGURES

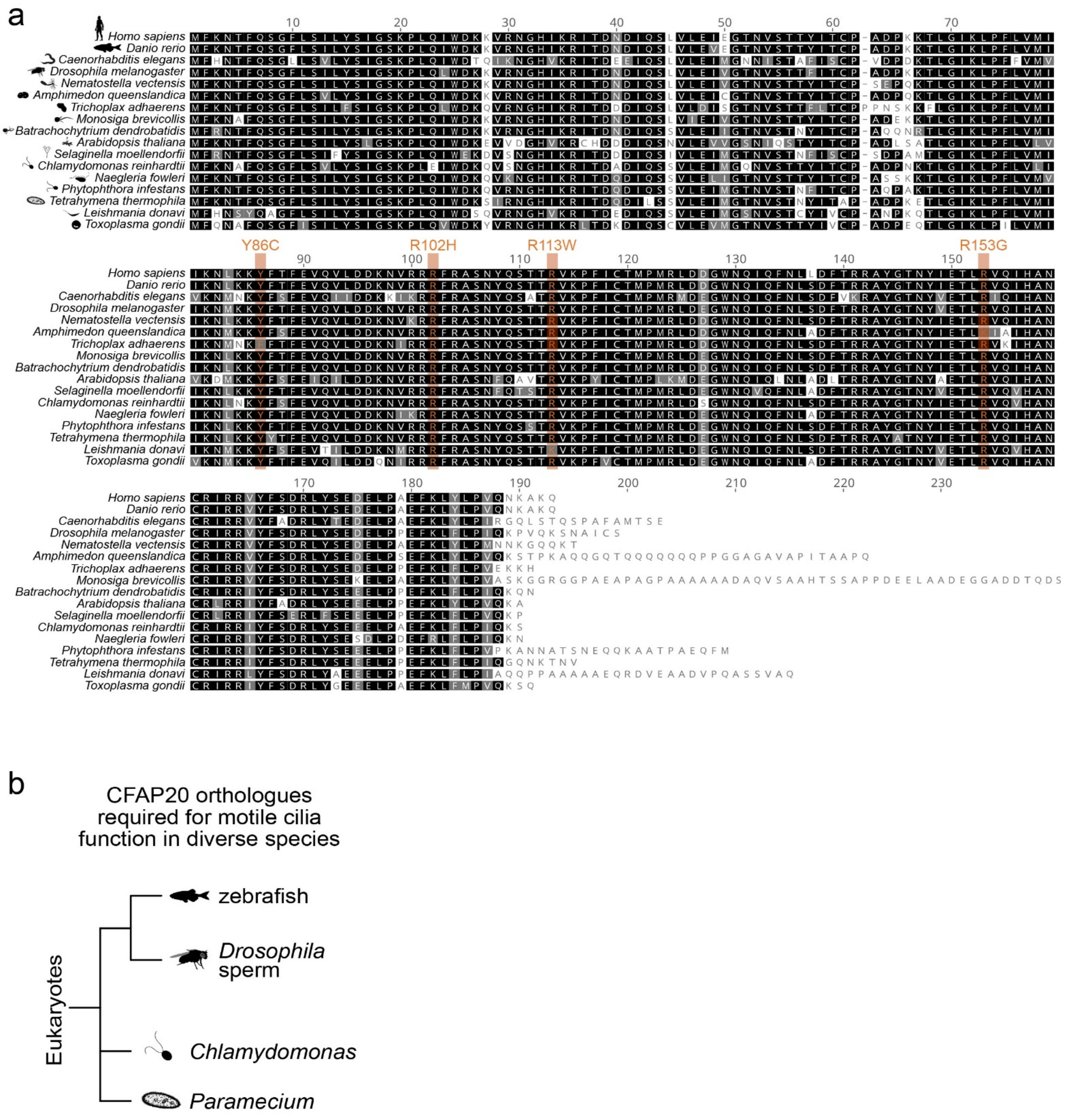

Supplementary Figure 1. **CFAP20 is highly conserved across eukaryotes.** Accompanies Figs. 2, 3 and 5. **(a)** Multiple sequence alignment of CFAP20 orthologues from a broad taxonomic representation of eukaryotes. All species are ciliated, except for *Arabidopsis thaliana*. Evolutionarily conserved residues are highlighted in black (identical) or gray (similar). Orange is used to highlight point mutations uncovered in retinitis pigmentosa, and each is at a deeply conserved residue shared by most unicellular organisms and diverse animals. The *T. adhaerens* and *S. moellendorfii* icons were created by Oliver Voigt and Ville Koistinen (vectorized by T. Michael Keesey), respectively, under the CC-BY-SA 3.0 license

(https://creativecommons.org/licenses/by-sa/3.0/). (**b)** Genetic manipulations have shown a deeply conserved requirement for *CFAP20* in motile cilia. Diverse organisms investigated include unicellular ciliates, unicellular algae, and metazoans including invertebrate cells (*Drosophila* sperm) & vertebrate animals (zebrafish). See references[15, 16], and herein zebrafish data in **Fig. 2**.

5’ RACE

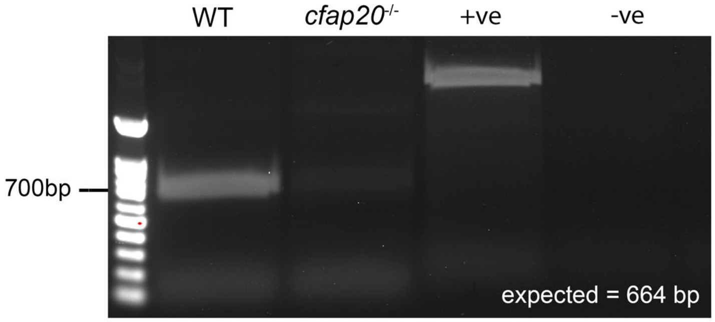

Supplementary Figure 2. 5’ RACE resolves no evidence of alternative transcripts in *cfap20*$^{-/-}$ homozygotes. 5’ rapid amplification of cDNA ends (RACE) analysis using a single reverse primer in exon 6 of zebrafish *cfap20*. Only a single major product was produced from templates of WT and *cfap20*$^{-/-}$ homozygotes at the expected size. Sequncing confirmed that these bands were WT and mutant *cfap20* respectively. SMARTer® RACE 5’/3’ Kit (Takara Bio) positive and negative controls were included. Single technical replicate from 30 pooled embryos. Source data are provided as a Source Data file.

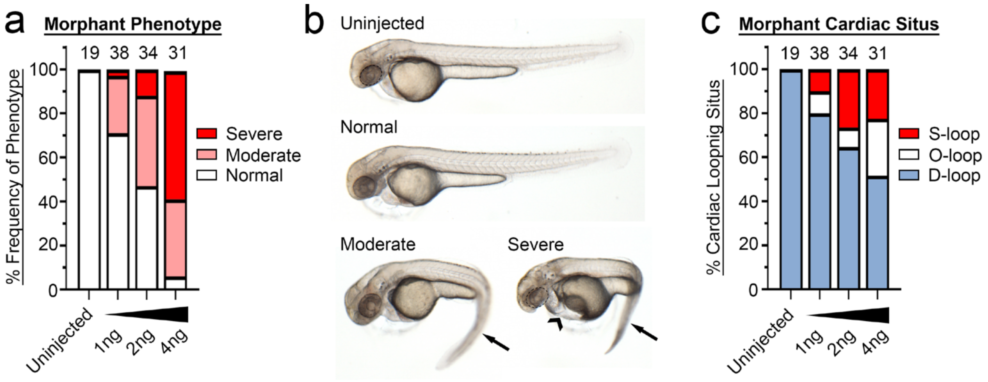

Supplementary Figure 3. Optimizing *cfap20* knockdown shows dose-responsive motile ciliopathy phenotypes in zebrafish larvae. Accompanies Figure 2. (**a**) Increasing morpholino dosage correlated with increased frequency of embryos scored as moderate and severe; n = 2 clutches, total embryo number above each bar. (**b**) Brightfield micrographs showing representative phenotypes at 48 hpf of uninjected controls, phenotypically normal, moderate, and severely affected morphants (arrow – ventral body curvature, chevron – pericardial oedema, dotted ellipse highlighting the disparity in eye size between uninjected and severe). (**c**) Increasing morpholino dosage correlated with reduced frequency of normal, D-looped hearts at 48 hpf, and increase in O-loop and S-looped hearts; n = 2 clutches, total embryo number above each bar. Source data are provided as a Source Data file.

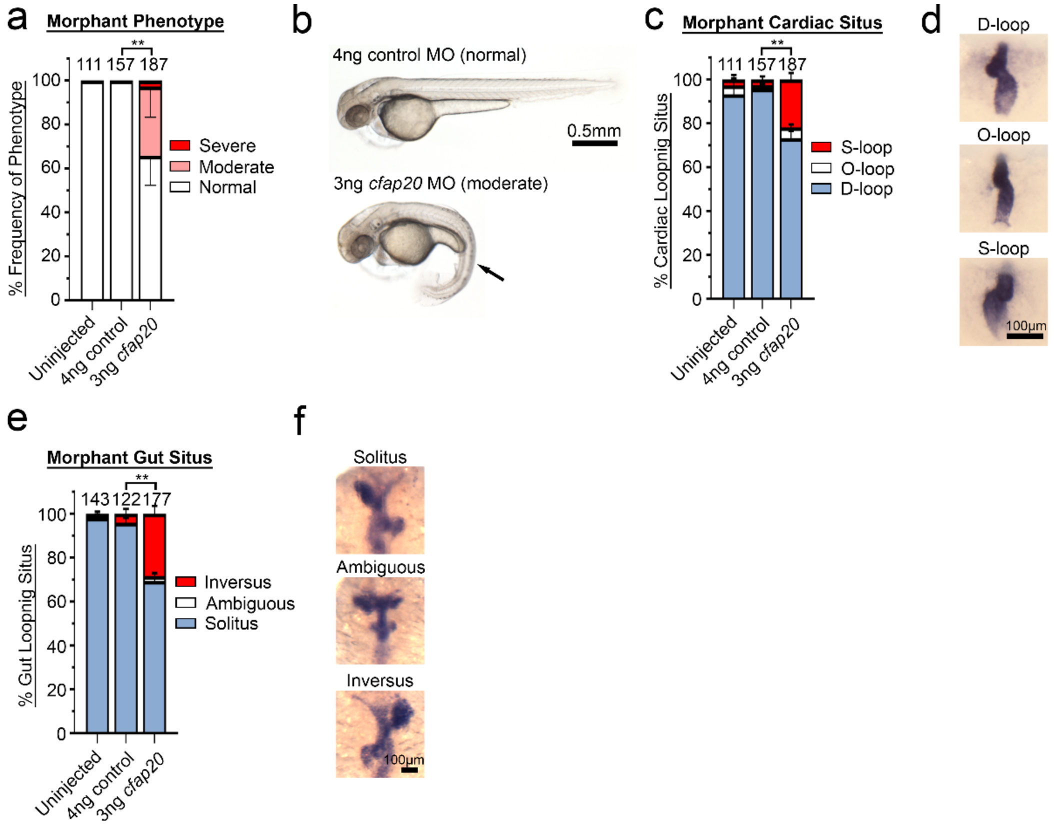

Supplementary Figure 4. *cfap20* knockdown phenocopies mutants and produces archetypal motile cilia phenotypes in zebrafish larvae. Accompanies Fig. 2. (**a**) Quantification of morphant embryos scored as phenotypically normal, moderate or severe at 48 hpf (n = 6 clutches, total embryo number above each bar; One-way ANOVA and Dunnett's *post hoc* test; 4ng control vs. uninjected P = 0.999; 4ng control vs. 3ng *cfap20* P = 0.0130). (**b**) Brightfield micrographs showing representative phenotypes at 48 hpf of 4ng control MO and 3ng *cfap20* MO injected embryos (arrow – ventral body curvature). (**c**) Quantification of cardiac looping situs at 48 hpf (n = 6 clutches, total embryo number above each bar; one-way ANOVA and Dunnett's *post hoc* test; 4ng control vs. uninjected P = 0.7597; 4ng control vs. 3ng *cfap20* P = 0.0001). (**d**) Ventrally imaged wholemount *in situ* hybridisation (*myl7*) micrographs of 3ng *cfap20* morphants with D-loop, O-loop and S-loop heart. (**e**) Quantification of gut situs at 48 hpf (n = 6 clutches, total embryo number above each bar; ; one-way ANOVA and Dunnett's *post hoc* test; 4ng control vs. uninjected P = 0.7520; 4ng control vs. 3ng *cfap20* P = 0.0001). (**f**) Dorsally imaged wholemount *in situ* hybridisation (*foxa3*) micrographs of 3ng *cfap20* morphants with *solitus*, *ambiguous* and *inversus* gut situs. Source data are provided as a Source Data file.

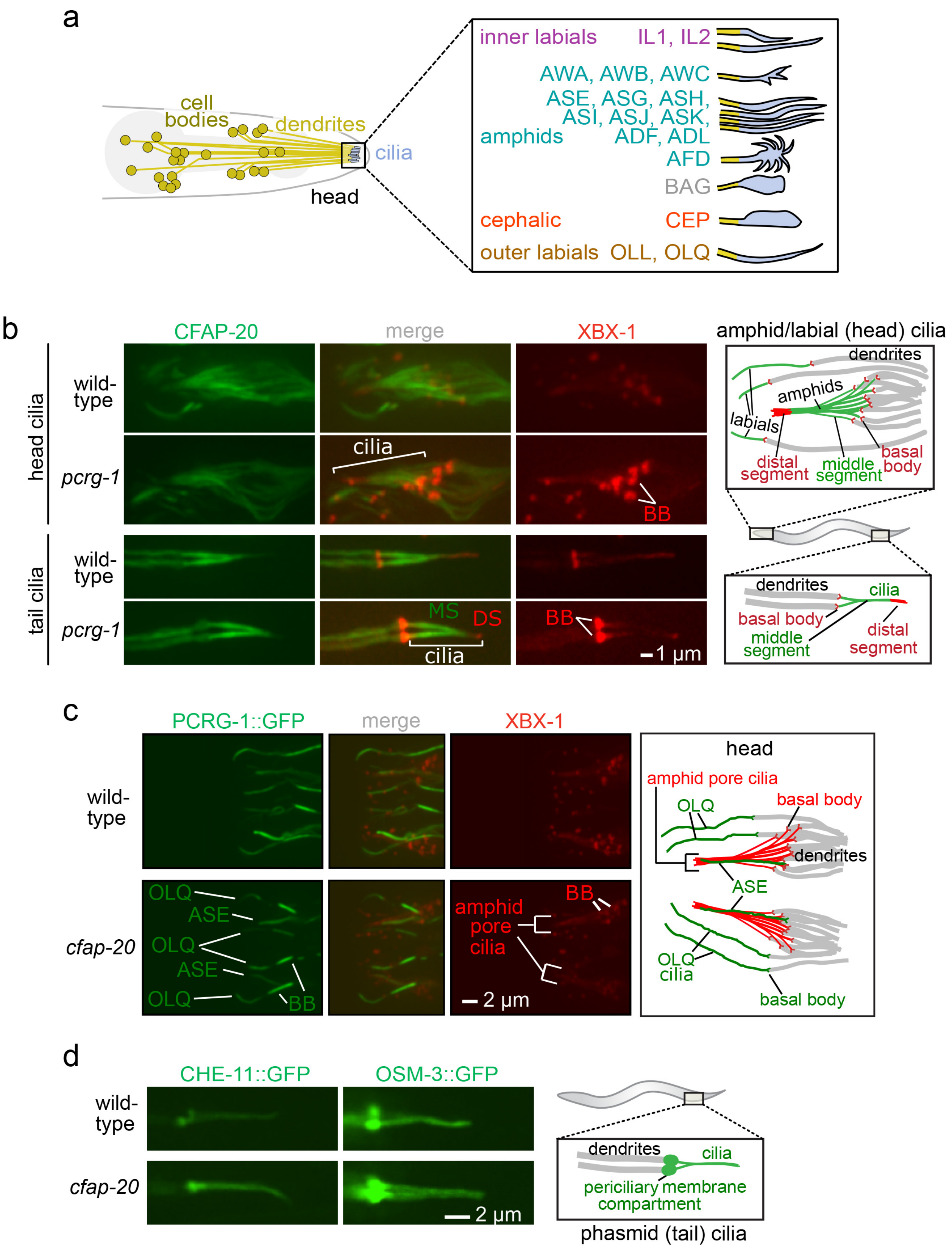

Supplementary Figure 5. *C. elegans* CFAP-20 and PCRG-1 localisation to cilia is independent, and *cfap-20* mutants retain a full complement of neuronal cilia. (**a**) Schematic showing the localisation of ciliated head-localised neurons and their form. (**b**) GFP-tagged CFAP-20 localises normally to the proximal segment of *pcrg-1* mutant cilia. (**c**) GFP-tagged PCRG-1 localises normally to OLQ and ASE cilia in a strain

lacking CFAP-20. The XBX-1::tdTomato reporter marks the basal body (BB) and entire ciliary axoneme. (**d**) The *cfap-20* null mutant displays seemingly intact cilia (phasmid cilia shown) based on two IFT reporters, OSM-3::GFP and CHE-11::GFP.

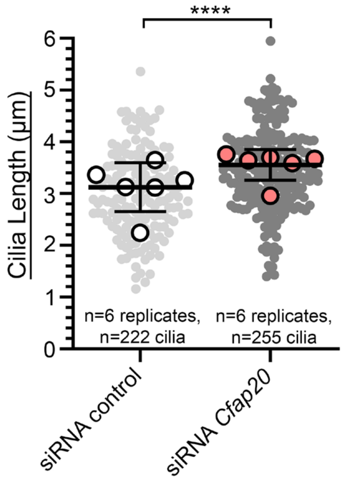

Supplementary Figure 6. *Cfap20* knockdown increases mammalian non-motile cilia length. Accompanies Fig. 3. *Cfap20* siRNA treatment increases cilia length in NIH 3T3 cells compared to non-targeting control siRNA. Total cilia measurement number and n-value below graph. All data points were analysed via two-tailed unpaired T-Test assuming unequal variance (F-test P=0.792644; error bars = SEM) using GraphPad Prism software. This test returned a P-value of 5.27×10^{-8}. Normal distribution of the data was confirmed using a QQ plot. Mean values of ciliary length were 3.003 μM for siRNA Control cells and 3.462 μM for siRNA *CFAP20.* Source data are provided as a Source Data file.

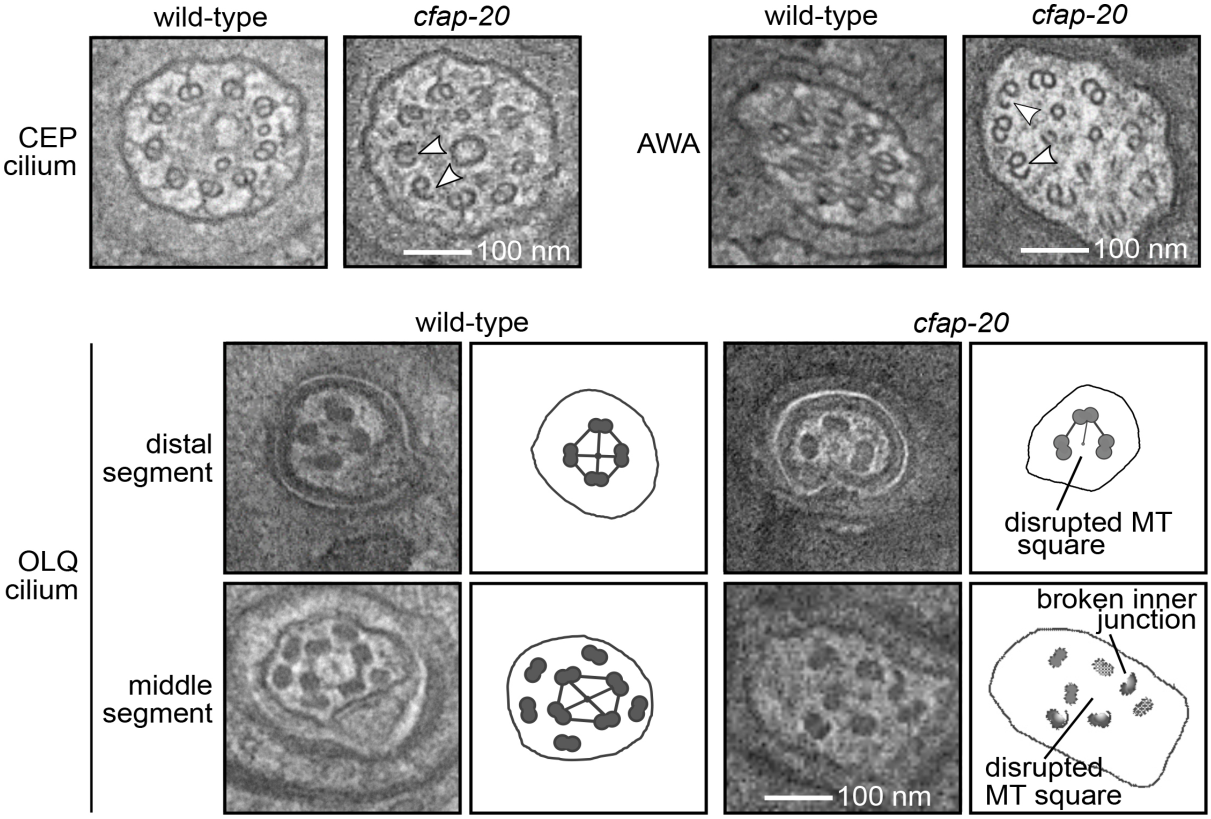

Supplementary Figure 7. Loss of *C. elegans* CFAP-20 results in inner junction defects in different ciliary types, and ultrastructural defect of OLQ cilia. Transmission Electron Micrographs of *C. elegans* wild-type and a *cfap-20* mutant in the head region, where various classes of cilia are present. Shown are cilia cross sections from CEP and AWA neuron cilia. Other cilia from BAG, IL1, and IL2 neurons in *cfap-20* mutant animals also show B-tubule defects similar to the amphid cilia shown in Fig. 3c. OLQ cilia, which are specialised mechanosensors with a unique morphology, show missing doublets and disrupted architecture in the *cfap-20* that is similar to the previously published *pcrg-1* mutants[37].

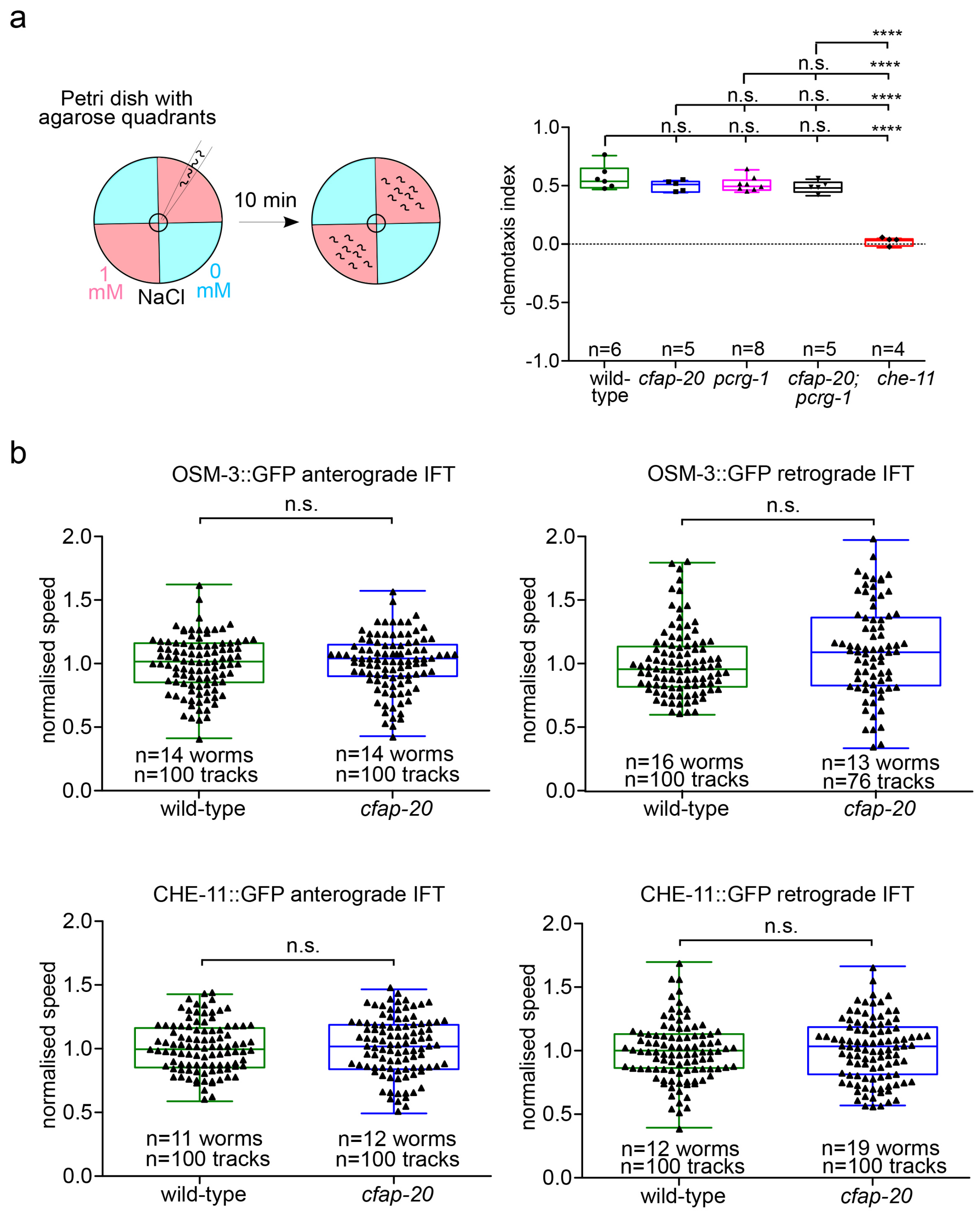

Supplementary Figure 8. *C. elegans cfap-20* mutants show normal salt-sensing and intraflagellar transport. (**a**) *C. elegans cfap-20* mutants show normal salt-sensing behaviours as measured by chemotaxis assays for NaCl. The control mutant strain *che-11*, impaired in IFT and lacking cilia, does not show a preference for any salt concentrations (one-way ANOVA and Tukey's test; *P* values: WT vs cfap-20 = 0.4209; WT vs pcrg-1 = 0.5197; WT vs cfap-20;pcrg-1 = 0.3169; WT vs che-11 = 0.0001; cfap-20 vs pcrg-1 = 0.996; cfap-20 vs cfap-20;pcrg-1 = 0.9997; cfap-20 vs che-11 = 0.0001; pcrg-1 vs cfap-20;pcrg-1

= 0.9773; pcrg-1 vs che-11 = 0.0001; cfap-20;pcrg-1 vs che-11 = 0.0001). In a chemotaxis assay, animals are placed in the middle of plates with alternating low- and high-NaCl quadrants (0 mM and 1 mM, respectively) and chemotaxis index scoring after 10 min. (**b)** IFT speeds are not dramatically altered in the *cfap-20* mutant. Anteriorgrade and retrograde IFT speed measurements of OSM-3::GFP (IFT-B reporter) and CHE-11::GFP (IFT-A reporter) are similar between wild-type and *cfap-20* mutants (unpaired t-test; *P* values: OSM-3 WT vs cfap-20 anterograde = 0.6408; retrograde = 0.0313; CHE-11 WT vs cfap-20 anterograde = 0.6936; retrograde = 0.6467). All box plots represent minima, 25th percentile, median, 75th percentile, maxima. Source data are provided as a Source Data file.

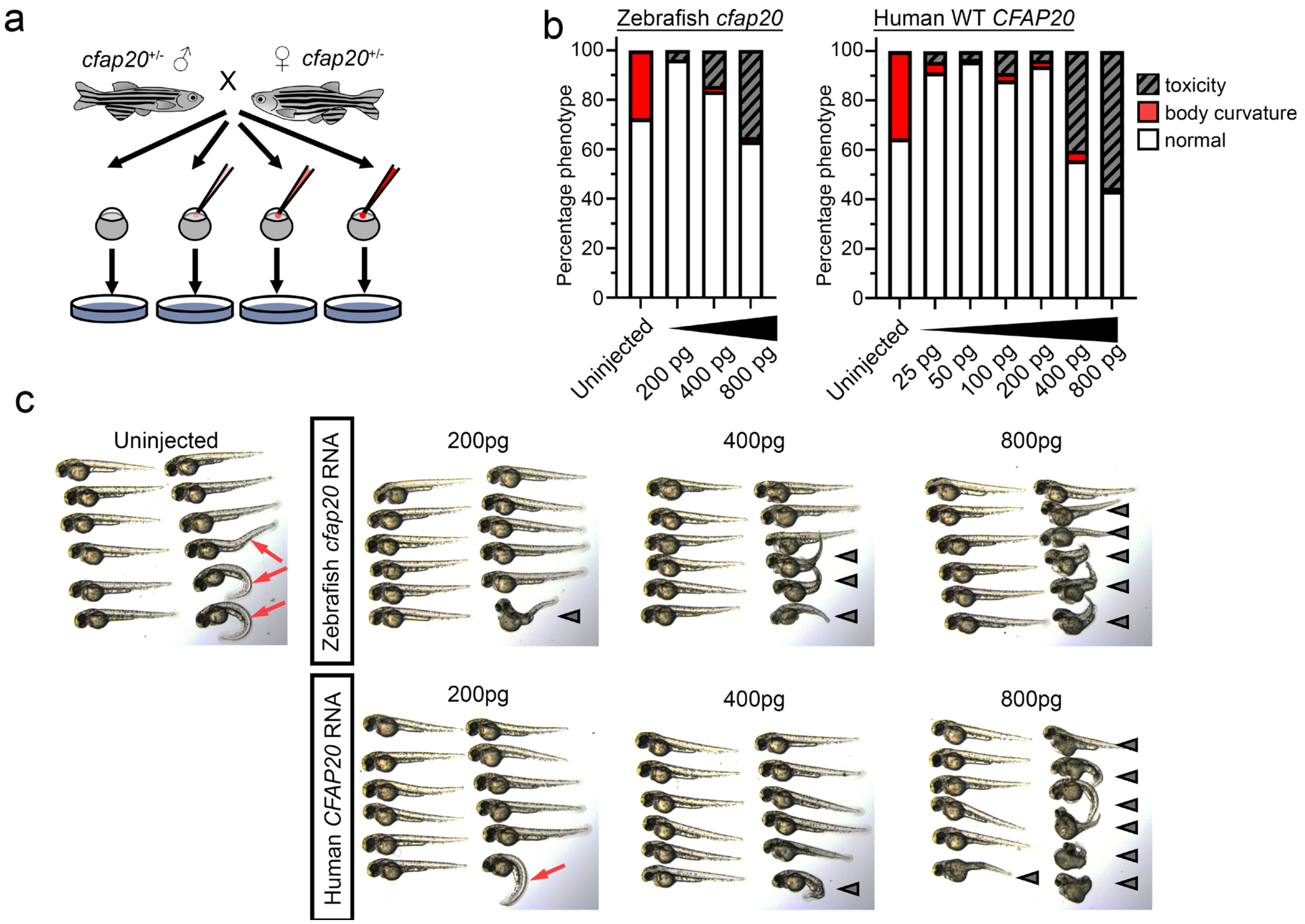

Supplementary Figure 9. *cfap20*$^{-/-}$ homozygote body curvature can be rescued by either human or zebrafish *CFAP20* mRNA overexpression. (a) Schematic of the rescue experiment protocol. Embryos from a *cfap20*$^{+/-}$ heterozygote incross were injected with *CFAP20* mRNA at different concentrations to generate dose response data. Experimenter was blinded to treatment prior to scoring outocomes. Mendelian ratios (25%) of progeny are expected to exhibit the *cfap20*$^{-/-}$ phenotype (e.g. body curvature in panel B), and this percentage will be reduced if the mRNA rescues the phenotype. 50 embryos per clutch were left uninjected to ensure >80% fitness **(b, c)** *CFAP20* homologs form either zebrafish or human were able to rescue the mutant phenotype, with respect to motile cilia phenotype, when mRNA was delivered at 200 pg dose. Larger doses led to maldevelopment ("toxicity"). Human *CFAP20* mRNA was tested at lower doses to prepare for comparison to patient variants, and low doses such as 25pg of mRNA were able to substantially rescue phenotypes. For all groups >50 embryos were scored by blinded observers at 48 hours post-fertilizatin (hpf) based on prevalence of body curvature defects, and noting signs of mRNA toxicity. Toxicity was observed at < 10% prevalence when rescuing with 200 pg of either zebrafish *cfap20* or human WT *CFAP20* mRNA (arrowheads). Furthermore, body curvature defects (arrows) had <10% prevalence at ≤ 200 pg *CFAP20* doses. Reults from delivery of control mRNA (mCherry) is presented in Figure 6c,d. This control, combined with reduce rescue in mutant CFAP20 (Figure 6c,d) suggest the rescue is the result of replacing *CFAP20*. Together, the data strongly support that the phenotype is caused specifically by *cfap20* mutation. Ocular phenotypes associated with *cfap20* disruption appear too late in development for mRNA rescue to be a practical approach. Source data are provided as a Source Data file.

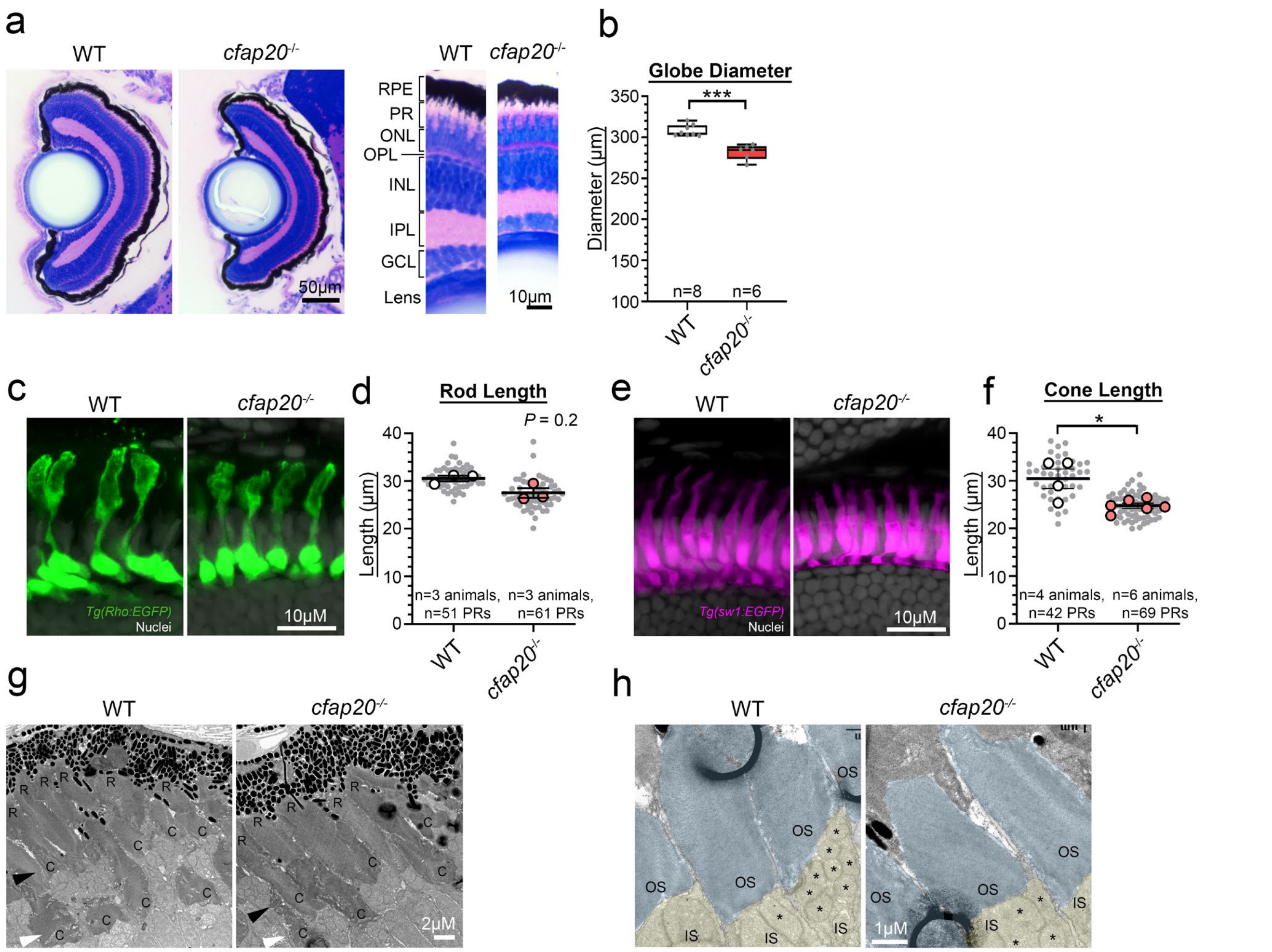

Supplementary Figure 10. Development of the neuroretina is grossly normal in early larval *cfap20*-/- zebrafish. (**a**) Low and high magnification histology of 7 days post-fertilization (dpf) *cfap20*-/- homozygote with all neuroretinal layers marked. (**b**) Globe diameter quantification (n-value below data, two-tailed unpaired T-test, *P* <0.0001). (**c,d**) Maximum intensity projection through the photoreceptor layer of Tg(-3.7rho:EGFP)kj2 larvae (7 dpf) revealed normal rod histology measured as length in *cfap20*-/- mutants (n = 3; error bars = SEM; total number of rods measurements below data; two-tailed unpaired T-test, *P* = 0.0612). (**e,f**) Maximum intensity projection through photoreceptor layer of Tg(5.5opn1sw1:EGFP)kj9 larvae (7 dpf) revealed shortened cone histology in *cfap20*-/- mutants (n = 4, 6; total number of cones measurements below data; error bars = SEM; two-tailed unpaired T-test, *P* = 0.0119). **(g)** TEM micrographs through the retina revealed normal lamination of the photoreceptor layer with outer rods (R), intermediate blue / red / green cones (C, black arrowhead), and inner UV cones (C, white arrowhead). **(h)** At higher magnification, the outer segments (blue) of *cfap20*-/- homozygotes possess normally stacked discs at 7 dpf (three replicates per condition performed). OS = outer segment, IS = inner segment, * = mitochondria, RPE = retinal pigment epithelium, PR = photoreceptor, ONL = outer plexiform layer, OPL = inner plexiform layer, INL = inner nuclear layer, IPL = inner plexiform layer, GCL = ganglion cell layer. Source data are provided as a Source Data file.

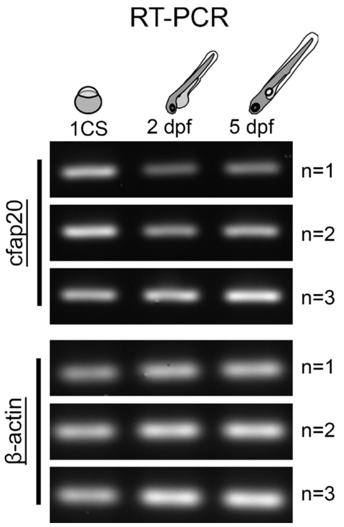

Supplementary Figure 11. Zebrafish *cfap20* mRNA is maternally inherited. Reverse-transcription PCR demonstrates that *cfap20* mRNA is maternally inherited at the 1 cell stage (CS) and being transcribed at both 2 and 5 days post fertilisation (dpf). n = 3 biological replicates. β-actin primers utilised as a template loading control. Source data are provided as a Source Data file.

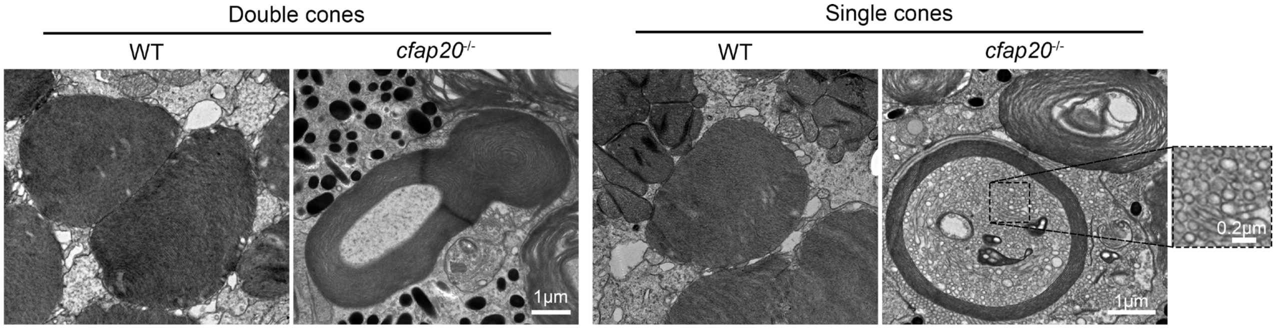

Supplementary Figure 12. *cfap20*$^{-/-}$ homozygotes display dysmorphic photoreceptor outer segment discs filled with vesicular-like structures. TEM cross-sections through the photoreceptor outer segment of 4 mpf zebrafish reveals that instead of compact, flattened discs, *cfap20*$^{-/-}$ homozygote discs are dysmorphic and appear "unwound". The unwound outer segments are filled by vesicle-like structures (insert). Three replicates per condition were performed.

TABLES

Supplementary Table 1. *C. elegans* strains used in this study.

Strain	Genotype
Bristol N2	wild-type
PD1074	wild-type
MX130	che-11(e1810)
MX450	pcrg-1(tm2597)
PHX627	cfap-20(syb627)
MX3021	cfap-20(syb627); pcrg-1(tm2597)
MX1578	N2; nxEx65[cfap-20::gfp + osm-5p::xbx-1tdTomato + rol-6(su1006)]
MX3087	pcrg-1(tm2597); nxEx65[cfap-20::gfp + osm-5p::xbx-1tdTomato + rol-6(su1006)]
MX1059	N2; nxIs60[pcrg-1::gfp];nxEx651[xbx-1::tdTomato + rol-6(su1006)]
MX3088	cfap-20(syb627); nxIs60[pcrg-1::gfp];nxEx651[xbx-1::tdTomato + rol-6(su1006)]
MX60	N2; myEx10[che-11::gfp + rol-6(su1006)]
MX255	N2; ejEx1[osm-3::gfp + rol-6(su1006)]
MX3084	cfap-20(syb627); pcrg-1(tm2597); myEx10[che-11::gfp + rol6(su1006)]
MX3090	cfap-20(syb627); ejEx1[osm-3::gfp + rol-6(su1006)]
MX3091	pcrg-1(tm2597); ejEx1[osm-3::gfp + rol-6(su1006)]
MX3074	cfap-20(syb627); myEx10[che-11::gfp + rol6(su1006)]
MX512	pcrg-1(tm2597); myEx10[che-11::gfp + rol6(su1006)]

Supplementary Table 2. *CFAP20* variants identified across five families.

Accession	DNA	Protein	SNP ID	gnomAD MAF	SIFT tolerated?	Polyphen-2 Damaging?	PROVEAN	Mutation-Taster
NM_013242.3	c.305G>A	p.(Arg102His)	rs1487548809	1.06E-05	No	Probably (1.00)	Deleterious	Disease Causing
NM_013242.3	c.257A>G	p.(Tyr86Cys)	rs1377392375	3.98E-06	No	Probably (0.994)	Deleterious	Disease Causing
NM_013242.3	c.337C>T	p.(Arg113Trp)	rs767845554	1.41E-05	No	Probably (1.00)	Deleterious	Disease Causing
NM_013242.3	c.397del	p.(Gln133Serfs*5)	rs755182323	3.98E-06	-	-	-	-
NC_000016.10 (CFAP20_v002)	c.164+1 G>A	-	rs767928662	3.98E-06	-	-	-	-
NM_013242.3	c.457A>G	p.(Arg153Gly)	-	-	No	Probably (0.836)	Deleterious	Disease Causing

Supplementary Table 3. Summary of clinical findings.

**Family-Individual ID**	**F1:1**	**F2:1**	**F3:1**	**F4:1**	**F4:2**	**F4:3**
Left eye BCVA	LP	NA	20/20	20/20	20/40	20/70
Right-eye BCVA	LP	NA	20/20	20/40	20/40	20/200
Reduced Visual Fields	+	+	+	+	+	+
Myopia	+	+	+	+	+	+
Night Blindness	+	+	+	+	+	+
RPE loss/Bone spicules	+	+	+	+	+	+
Abnormal/Reduced ERG	+	NA	+	+	+	+
Motor Delays	-	-	-	+	+	+
Learning Disabilities	-	-	-	+	+	+
Seizures	-	-	-	-	-	+
Reproductive Issues	NA	NA	NA	+	+	+

BCVA = Best-Corrected Visual Acuity**, ERG** = Electroretinogram **LP** = Light perception **NA** = Not Assessed

Supplementary Table 4. Zebrafish key resources.

Zebrafish strains		
Name	Source	Zfin ID
AB	ZIRC	ZDB-GENO-960809-7
Tg(-3.7rho:EGFP)kj2	Hamaoka, 2002 *PMID: 12395387*	ZDB-ALT-060830-4
Tg(-5.5opn1sw1:EGFP)kj9	Takechi, 2003 *PMID: 14550552*	ZDB-ALT-080227-1

Antibodies		
Name	Source	Zfin ID / Cat. #
Mouse anti-arrestin 3a; zpr-1	ZIRC; PMID: 2401210	ZDB-ATB-081002-43
Unknown epitope of rod photoreceptors; 4C12	Fadool lab; PMID: 16303977	ZDB-ATB-090506-2
Cleaved caspase-3	Cell Signalling Technology	#9661
rabbit anti-GFP antibody	Invitrogen	A-11122
Alexa Fluor 488 Chicken anti-Rabbit	Invitrogen	A-21441
Alexa Fluor 555 Donkey anti-Mouse	Invitrogen	A-31570
TO-PRO™-3 Iodide	Invitrogen	T3605

Oligonucleotides		
Name	Source	Zfin ID / Sequence
MO1-cfap20	Gene-Tools; PMID: 24574454	ZDB-MRPHLNO-181030-2
cfap20 genotyping primer fwd	IDT	GCACCACATACATCACGTGTCC
cfap20 genotyping primer rev	IDT	AGAAAAGCTCCAAGAGAAACACACA
cfap20 qPCR primer fwd	IDT	ACCCCTGCAGATATGGGACA
cfap20 qPCR primer rev	IDT	GCTTACGTTGGTTCCCTCCA
gapdh qPCR primer fwd	IDT; PMID: 33299975	CCACCCCCAATGTCTCTGTT

gapdh qPCR primer rev	IDT; PMID: 33299975	TCATACTTGGCAGGTTTCTCAA
B-actin qPCR primer fwd	IDT; PMID: 23523635	CGGACAGGTCATCACCATTG
B-actin qPCR primer rev	IDT; PMID: 23523635	GATGTCGACGTCACACTTCA
sgRNA_constant_oligo	IDT	AAAAGCACCGACTCGGTGCCACTTTTTCAAGTTGATAACGGACTAGCCTTATTTTAACTTGCTATTTCTAGCTCTAAAAC
gRNA_cfap20_1	IDT	ATTTAGGTGACACTATACGAATGTGAAATACTTCTTGGTTTTAGAGCTAGAAATAGCAAG
gRNA_cfap20_1	IDT	ATTTAGGTGACACTATAGGAAAGGAAGCTTGATGCCGGTTTTAGAGCTAGAAATAGCAAG
5' RACE primer	IDT	GATTACGCCAAGCTTCGAGAAGCGTGGAGAAACCCTGCCG

Chemicals		
Name	Source	Catelogue #
UltraPure™ Glycerol	ThermoFisher Scientific	15514011
Permount™ Mounting Medium	Fisher Scientific	SP15-100
MEGAscript™ SP6 Transcription Kit	ThermoFisher Scientific	AM1330
EnGen® Spy Cas9 NLS	NEB	M0646M
pGEM®-T Vector	Promega	A3610
GoTaq® G2 DNA Polymerase	Promega	M7848
Boric acid	Millipore Sigma	B7901
Sodium Hydroxide	Millipore Sigma	S5881
SP6 RNA Polymerase	Millipore Sigma	10810274001
digoxigenin-UTP labelling mix	Millipore Sigma	11277073910
TRIzol	ThermoFisher Scientific	15596026
Chloroform	ThermoFisher Scientific	C298-1
Proteinase K	Millipore Sigma	P2308
Paraformaldehyde	Millipore Sigma	158127
Anti-digoxigenin-AP Fab fragments	Millipore Sigma	11093274910

NBT/BCIP	Millipore Sigma	11681451001
Technovit® 7100 resin	Technovit	64709003
Permount™ Mounting Medium	Fisher Scientific	SP15
Tissue-Tek® O.C.T.	Sakura	4583
ProLong™ Gold Antifade Mountant	ThermoFisher Scientific	P36930
Tricaine Methanesulfonate	Millipore Sigma	E10521

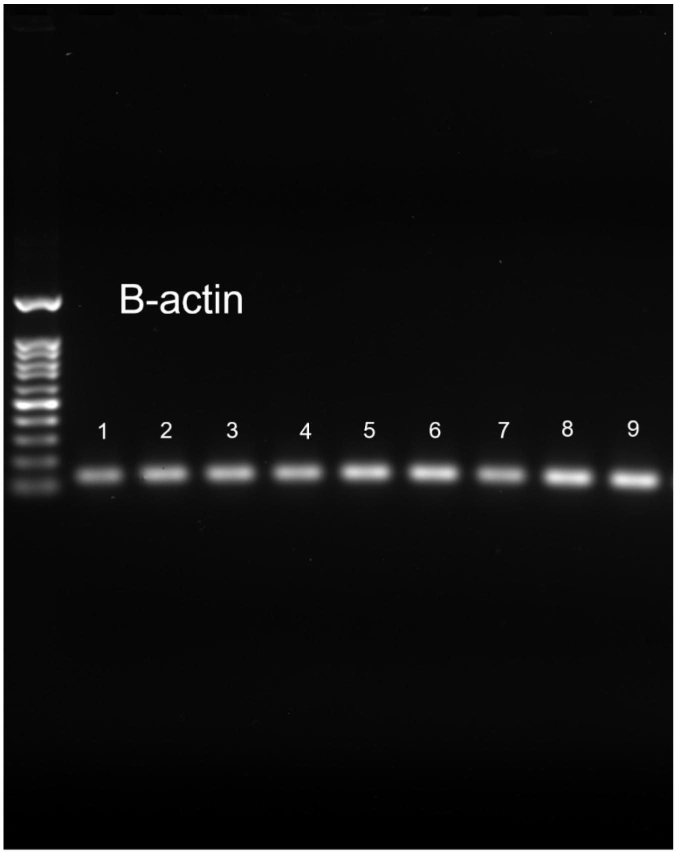

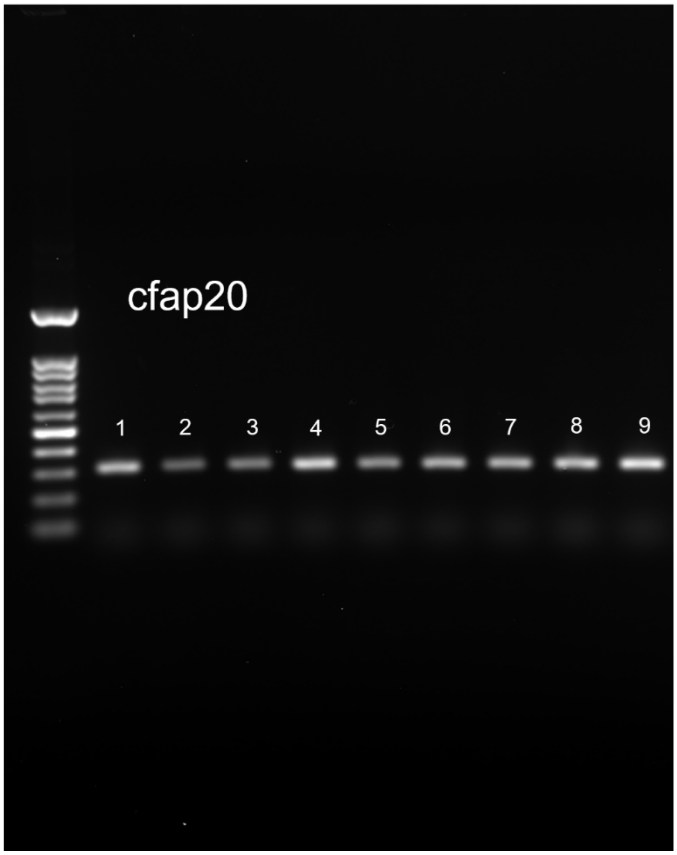

Uncropped, raw gel for Supplementary Figure 11.

	n =	stage / timepoint
lane 1	1	1 cell stage
lane 2	1	2 dpf
lane 3	1	5 dpf
lane 4	2	1 cell stage
lane 5	2	2 dpf
lane 6	2	5 dpf
lane 7	3	1 cell stage
lane 8	3	2 dpf
lane 9	3	5 dpf
