# Supplementary material for: The inner junction protein CFAP20 functions in motile and non-motile cilia and is critical for vision
Source: Nat Commun. 2022 Nov 3;13:6595. doi: 10.1038/s41467-022-33820-w (PMC9633640; doi:10.1038/s41467-022-33820-w)
Supplement: Supplementary file 2 — Reporting Summary [file 41467_2022_33820_MOESM2_ESM.pdf]

nature portfolio

Corresponding author(s): W. Ted Allison, Ping Yee Billie Au, Ian M. MacDonald, Gavin Arno, Michel R. Leroux

Last updated by author(s): Aug 29, 2022

Reporting Summary

Nature Portfolio wishes to improve the reproducibility of the work that we publish. This form provides structure for consistency and transparency in reporting. For further information on Nature Portfolio policies, see our Editorial Policies and the Editorial Policy Checklist.

Statistics

For all statistical analyses, confirm that the following items are present in the figure legend, table legend, main text, or Methods section.

n/a	Confirmed	
☐	☒	The exact sample size (*n*) for each experimental group/condition, given as a discrete number and unit of measurement
☐	☒	A statement on whether measurements were taken from distinct samples or whether the same sample was measured repeatedly
☐	☒	The statistical test(s) used AND whether they are one- or two-sided<br>*Only common tests should be described solely by name; describe more complex techniques in the Methods section.*
☒	☐	A description of all covariates tested
☐	☒	A description of any assumptions or corrections, such as tests of normality and adjustment for multiple comparisons
☐	☒	A full description of the statistical parameters including central tendency (e.g. means) or other basic estimates (e.g. regression coefficient) AND variation (e.g. standard deviation) or associated estimates of uncertainty (e.g. confidence intervals)
☐	☒	For null hypothesis testing, the test statistic (e.g. *F*, *t*, *r*) with confidence intervals, effect sizes, degrees of freedom and *P* value noted<br>*Give P values as exact values whenever suitable.*
☒	☐	For Bayesian analysis, information on the choice of priors and Markov chain Monte Carlo settings
☒	☐	For hierarchical and complex designs, identification of the appropriate level for tests and full reporting of outcomes
☒	☐	Estimates of effect sizes (e.g. Cohen's *d*, Pearson's *r*), indicating how they were calculated

Our web collection on statistics for biologists contains articles on many of the points above.

Software and code

Policy information about availability of computer code

Data collection	For C. elegans<br>Transmission electron microscopy imaging was performed with the Tecnai T12 inbuilt FEI proprietary software<br>Images for body length analysis were acquired with Open lab software version 5.<br>All other images were generated with Volocity version 6.5.1<br>Length measurements were performed with ImageJ version 1.53e<br>Kymographs were generated with KymographClear version 1.0<br><br>For Mammalian Cell Experiments<br>Graphpad Prism v.9.2.0(332)<br>FIJI Open Source Image Software v.1.8.0<br><br>For zebrafish<br>Zeiss Zen 3.2 confocal imaging.<br>QCapture 5 brightfield capture.<br>InVivoVue 2.4 OCT Management Software for OCT.
Data analysis	For C. elegans<br>Statistics for gustatory plasticity were performed with SPSS version 25<br>All other worm statistics were calculated with Graphpad Prism version 5.0.1<br><br>For Mammalian Cell Experiments<br>Graphpad Prism - Statistical analysis and data representation<br>FIJI OPen Source Image Software - immunofluorescence analyses

For zebrafish
GraphPad Prism 9.0.2; Zeiss Zen 3.2; FIJI-win64; Slicer 4.11.20200930; Geneious 2021-03-12; Photoshop 22.4.3

For manuscripts utilizing custom algorithms or software that are central to the research but not yet described in published literature, software must be made available to editors and reviewers. We strongly encourage code deposition in a community repository (e.g. GitHub). See the Nature Portfolio guidelines for submitting code & software for further information.

Data

Policy information about availability of data

All manuscripts must include a data availability statement. This statement should provide the following information, where applicable:

- Accession codes, unique identifiers, or web links for publicly available datasets
- A description of any restrictions on data availability
- For clinical datasets or third party data, please ensure that the statement adheres to our policy

All data are available from the authors on request. The data for the variants identified in families 1-4 have been submitted to ClinVar (https://www.ncbi.nlm.nih.gov/clinvar/). The structure of the axonemal inner junction complex was obtained from the Protein Data Bank (6VE7). Zebrafish cfap20 gene structure information was obtained from ENSEMBL release GRCz11 (ENSDARG00000100514). Data accessibility information for the 100KGP is available online (www.genomicsengland.co.uk/join-a-gecip-domain). All data generated during this study are included in this article, its Supplement file and the Source Data file provided with this paper.

Field-specific reporting

Please select the one below that is the best fit for your research. If you are not sure, read the appropriate sections before making your selection.

☒ Life sciences ☐ Behavioural & social sciences ☐ Ecological, evolutionary & environmental sciences

For a reference copy of the document with all sections, see nature.com/documents/nr-reporting-summary-flat.pdf

Life sciences study design

All studies must disclose on these points even when the disclosure is negative.

Sample size

No predetermined sample-size calculations were performed. For adult zebrafish experiments, a sample size of at least 3 animals per condition was selected to ensure reproducibility whilst reducing animal usage. For larval zebrafish experiments at least 3 clutches of embryos were used to conform with best practices.

Data exclusions

For C. elegans
If worms moved during imaging, distorting maximum intensity projections of Z stacks, they were not analysed.
For roaming assays: Worms crawling off the plate or burying into the agar during the assay were excluded from analysis.
For lifespan assays: Worms crawling off the plate or burying into the agar during the assay were censored in accordance with Graphpad Prism recommendations for Kaplan-Meier survival analysis.
Worms with infections (burst vulva phenotype) were excluded from experiments.

For Zebrafish:
A clutch of embryos was discarded if fertility and fitness was not >70% at 24 hpf.
Sections were omitted if there were tears / artifacts after the corresponding staining protocol.
ERGs traces were excluded if high noise levels were present prior to light stimuli.

Replication

For C. elegans
All experiments (except TEM imaging) were performed at least twice, with distinct cohorts of worms, on different days.

For zebrafish
All experiments were replicated at least 3 times. For mutants a biological replicate was considered an individual animal. For morphants a clutch was used as a biological replicate.

Mammalian cell culture
All experiments were replicated in triplicate.

All results were successfully replicated.

Randomization

For C. elegans
Allocation of individual worms of one strain to experimental conditions was randomized - either through pipetting of worms (suspended in liquid).

For Zebrafish
All of the experiments involving microinjections (morpholino, mRNA injection) had treatments occur at the 1 cell stage, prior to genotype can be known; individuals were therefore randomly assigned to treatment groups. OCT and ERG recordings were performed on fish taken randomly from the same tank, and genotype was later assigned via PCR.

Blinding

For C. elegans
For behavioral experiments, strain plates were marked with a code, a few days before the experiment.
For image acquisition, no blinding was used. For analysis, image folders were duplicated and folder/strain names exchanged against a code, a few days before length measurement.
For length measurements, image folders were duplicated and blinded.

For zebrafish
Groups were blinded by colleagues prior to experiments. Unblinding was performed following data analysis.

For mammalian cell culture
Prior to cilia length data acquisition, immunofluorescence images were obtained by Dr. Doucette unblinded as no data acquisition was taking place at this time, simply image collection. Obtained images were blinded by Dr. Chrystal and returned to Dr. Doucette for cilia length determination. The subsequent data was then unblinded prior to statistical analyses.

Half life assays were not blinded. This was due to the nature of the timepoint acquisitions, and loading of gels/Western blotting. All data was normalized to loading controls to ensure data integrity and consistency.

Reporting for specific materials, systems and methods

We require information from authors about some types of materials, experimental systems and methods used in many studies. Here, indicate whether each material, system or method listed is relevant to your study. If you are not sure if a list item applies to your research, read the appropriate section before selecting a response.

Materials & experimental systems

n/a	Involved in the study
☐	☒ Antibodies
☐	☒ Eukaryotic cell lines
☒	☐ Palaeontology and archaeology
☐	☒ Animals and other organisms
☐	☒ Human research participants
☒	☐ Clinical data
☒	☐ Dual use research of concern

Methods

n/a	Involved in the study
☒	☐ ChIP-seq
☒	☐ Flow cytometry
☒	☐ MRI-based neuroimaging

Antibodies

Antibodies used

Primary Antibodies
Rabbit Anti-β-actin. Distributor: Santa Cruz Biotechnology, CA, USA, Cat #: sc-69879
Mouse Anti-Myc Tag: Distributor: Cell Signalling Technology. Cat #: 2276S
Mouse Anti-Acetylated Tubulin clone 6-11B-1 Distributor: Sigma-Aldrich Cat #: T7451
Mouse anti-arrestin 3a zpr-1; ZIRC; ZDB-ATB-081002-43
Unknown epitope of rod photoreceptors 4C12; ZIRC; ZDB-ATB-090506-2
rabbit anti-GFP antibody; Invitrogen; A-11122
Cleaved Caspase-3; Cell Signaling Technology; #9661

Secondary Antibodies:
Goat Anti-Mouse IgG Polyclonal Antibody (IRDye® 800CW) Distributor: LI-COR Biosciences NE, USA. Cat #:926-32210
Goat Anti-Rabbit IgG Polyclonal Antibody (IRDye® 680RD) Distributor: LI-COR Biosciences NE, USA. Cat #: 926-68071
Donkey anti-Rabbit IgG (H+L Alexa Fluor 555). Distributor: Invitrogen. Cat#: A31572
Alexa Fluor 488 goat anti-rabbit (ThermoFisher Scientific A-11008)
Alexa Fluor 488 Chicken anti-Rabbit; Invitrogen; A-21441
Alexa Fluor 555 Donkey anti-Mouse; Invitrogen; A-31570
TO-PRO™-3 Iodide; Invitrogen; T3605

Validation

Mammalian cell culture
All antibodies contain validation statements on the associated manufacturer/distributor's website. In our hands, all antibodies were validated via Western blot analysis using over-expression lysates generated via chemical transfection of HEK293T cells with a vector (pcDNA3.1 with CMV promotor) with appropriate gene insert. Banding patterns were compared to manufacturer's specifications/validation statements to ensure correct size. In the case of acetylated tubulin, this antibody was used to visualize primary cilia in NIH3T3 cell lines, a commonly used marker for cilia, data is presented in the manuscript.

Zebrafish
Antibodies were validated on WT tissues to ensure that they matched the immunofluorescent profile of previous publications.

Eukaryotic cell lines

Policy information about cell lines

Cell line source(s)	HEK293T and NIH3T3 both from ATCC
Authentication	Cell morphology and behavior were consistent with the cell identity.
Mycoplasma contamination	Mycoplasmia tests are performed every 6 months using PlasmoTest Mycoplasma Detection Kit (Supplier: Invivogen, Cat#: rep-pt1). All tests were negative.
Commonly misidentified lines (See ICLAC register)	No commonly misidentified lines used in this study

Animals and other organisms

Policy information about studies involving animals; ARRIVE guidelines recommended for reporting animal research

Laboratory animals	C. elegans does not fall under Canadian regulations for laboratory animals.<br>Only day one hermaphrodite worms were employed for experiments.<br>Bristol N2 or PD1074 background were used as wildtype/control.<br>Mutant and transgene strains used were<br>MX130 che-11(e1810), MX450 pcrg-1(tm2597), PHX627 cfap-20(syb627), MX3021 cfap-20(syb627); pcrg-1(tm2597),<br>MX1578 N2; nxEx65[cfap-20::gfp + osm-5p::xbx-1tdTomato + rol-6(su1006)],<br>MX3087 pcrg-1(tm2597); nxEx65[cfap-20::gfp + osm-5p::xbx-1tdTomato + rol-6(su1006)],<br>MX1059 N2; nxIs60[pcrg-1::gfp];nxEx651[xbx-1::tdTomato + rol-6(su1006)],<br>MX3088 cfap-20(syb627); nxIs60[pcrg-1::gfp];nxEx651[xbx-1::tdTomato + rol-6(su1006)],<br>MX60 N2; myEx10[che-11::gfp + rol-6(su1006)], MX255 N2; ejEx1[osm-3::gfp + rol-6(su1006)],<br>MX3084 cfap-20(syb627); pcrg-1(tm2597); myEx10[che-11::gfp + rol6(su1006)],<br>MX3090 cfap-20(syb627); ejEx1[osm-3::gfp + rol-6(su1006)], MX3091 pcrg-1(tm2597); ejEx1[osm-3::gfp + rol-6(su1006)]<br>MX3074 cfap-20(syb627); myEx10[che-11::gfp + rol6(su1006)], MX512 pcrg-1(tm2597); myEx10[che-11::gfp + rol6(su1006)]<br><br>Zebrafish (Danio rerio), were on AB strain background (including the transgenic animals Tg(-3.7rho:EGFP)kj2 & Tg(-5.5opn1sw1:EGFP)kj9) at 0-7 dpf, 1.5 mpf, 4 mpf and 8 mpf. Since mutants did not develop obvious sexual dimorphic traits, no distinction between sexes was performed in these experiments.
Wild animals	This study did not involve wild animals
Field-collected samples	This study did not involve samples collected from the field
Ethics oversight	For C. elegans no ethics oversight is required by the Canadian government.<br><br>Zebrafish lines were kept in accordance with the University of Alberta's Animal Care and Use Committee guidelines. Animal care protocols were approved by the University of Alberta Biosciences Animal Care Committee with protocol number AUP00000077.

Note that full information on the approval of the study protocol must also be provided in the manuscript.

Human research participants

Policy information about studies involving human research participants

Population characteristics	8 individuals, aged 31-70, 3 males, 5 females, with a clinical diagnosis of retinitis pigmentosa, from 4 unrelated families were recruited from Alberta and the UK.
Recruitment	Five affected individuals from three families were ascertained as part of the UK 100,000 genomes project (UK100KGP) (Turnbull et al., 2018), to which they were recruited to identify the genetic etiology of their retinal dystrophy. Family 4 was identified via clinical testing, and enrolled in research to investigate CFAP20 as a candidate gene, which lead to a connection to collaborators in the UK group.
Ethics oversight	Conjoint Health Research Ethics Board Calgary, Health Research Ethics Board University of Alberta, informed consent for WGS as part of the 100KGP was obtained in accordance with approval from the HRA Committee East of England – Cambridge South (REC Ref 14/EE/1112) and was approved by the Institutional Review Board and ethics committee of Moorfields Eye Hospital (MEH). This study was conducted in accordance with the criteria set out by the Declaration of Helsinki.

Note that full information on the approval of the study protocol must also be provided in the manuscript.
